# Hypertension in older adults in Africa: A systematic review and meta-analysis

**DOI:** 10.1371/journal.pone.0214934

**Published:** 2019-04-05

**Authors:** William Kofi Bosu, Siobhan Theresa Reilly, Justice Moses Kwaku Aheto, Eugenio Zucchelli

**Affiliations:** 1 Department of Public Health and Research, West African Health Organisation, Bobo-Dioulasso, Burkina Faso; 2 Division of Health Research, Faculty of Health & Medicine, Lancaster University, Lancaster, United Kingdom; 3 Department of Biostatistics, School of Public Health, University of Ghana, Legon, Accra, Ghana; Makerere University School of Public Health, UGANDA

## Abstract

**Background:**

Hypertension is the leading driver of cardiovascular disease deaths in Africa. Its prevalence is highest in older populations. Yet, this group has received little attention in many African countries. We conducted a systematic review and meta-analysis (PROSPERO registration: CRD42017056474) to estimate the prevalence of hypertension in older adults living in Africa.

**Methods:**

We searched grey literature and major electronic databases including PubMed and Embase for population-based studies and published between 1 January 1980 to 28 May 2018 reporting the prevalence of hypertension for adults aged ≥50 years living in Africa. We employed a random effects model to estimate the pooled prevalence across included studies.

**Findings:**

We screened 10,719 articles and retrieved 103 full-text articles to evaluate for inclusion in the review. Thirty-four unique studies providing 37 data points on 43,025 individuals in 15 African countries were analyzed. The prevalence of hypertension ranged from 22.3% to 90.0% from the individual studies while the overall pooled prevalence was 57.0% (95% CI 52%-61%). The prevalence was not statistically significantly different by sex, residence, or African sub-region. In individual studies, older age and overweight/obesity were independently associated with hypertension. Twenty-nine (78%) data points were deemed to be of low- or moderate-risk of bias. Eliminating high-risk bias studies made little difference to the pooled estimate of hypertension. Sensitivity analyses, omitting one study at a time, identified three studies with significant but relatively small impact on the pooled estimate. We observed substantial heterogeneity (I^2^ = 98.9%) across the studies which was further explored by meta-regression analyses. Overall, the GRADE assessment suggested moderate quality evidence in the results.

**Conclusion:**

The persistent high prevalence of hypertension among older adults in Africa, even in rural populations warrants more attention to the cardiovascular health of this group by public health authorities.

## Introduction

High systolic blood pressure is the leading risk for deaths in Africa. It resulted in nearly 900,000 deaths (10% of the total deaths on the continent) in 2016 and has increased by 82% since 1990 [1]. It is also responsible for more than half of first time acute stroke in Africa [2]. It is also a potentially modifiable risk factor for dementia, a disease of concern in Africa where ageing, stroke and other cardiovascular diseases are rising [3, 4]. A number of systematic analyses of hypertension in adults and in adolescents in Africa have recently been conducted [5–9]. The estimated pooled prevalence of hypertension is about 30.8% in Africa [5] and 30.0%-31.1% in Sub-Saharan Africa [6, 10]. Hypertension is now a significant problem in groups previously thought to be at low risk such as rural populations, poor households and young people [11–13]. It is a common cause of medical admissions in African hospitals [14]. With the ageing population and the rising urbanization and the attendant stress, westernized diet and low physical activity, high blood pressure will likely continue to rise [5, 15].

Hypertension increases steadily with increasing age. Its prevalence in the older adults in Africa is about two to four times that in younger adults [5, 10, 16]. Based on the Framingham study, it is estimated that about 90% of surviving normotensive persons aged 55 years will develop hypertension in their lifetime [17]. There is insufficient information on the cardiovascular health of older adults; a situation that partly contributes to the low attention paid to this group [18]. Only one systematic review of studies in older people with hypertension in Africa has been reported to date [19]. However, it did not review studies exclusively undertaken in older adults. Instead, it reviewed studies which sampled other age groups besides those aged 55 years and older. It analyzed 39 studies using the WHO STEPwise approach to surveillance [20] and 52 non-STEPS studies providing a total of 156 separate data contributions. The pooled prevalence was 55.2% [95% confidence interval (CI) 53.1–57.4] with little variation between younger and older age groups or over time. It is against this background that we undertook a systematic review and meta-analysis with the aim of estimating the prevalence of hypertension in adults aged 50 years or older in Africa and its distribution by demographic factors and over time. Our findings could inform the development of targeted policies aimed at improving cardiovascular health outcomes among this group.

The terms “elderly”, “aged” and “older persons”, are sometimes used interchangeably in the literature [21]. In most developed countries, presumably because of their higher life-expectancy, the cut-off age 65 years is used to define older adults. The United Nations conventionally uses 60 years as the threshold for older adults [22]. However, the 60 and 65 year-thresholds have been criticized as arbitrary and irrelevant for the African setting, where a World Health Organization (WHO)-project considers 50 years to be more appropriate for social and functional reasons [23]. Subsequently, several surveys in Africa have used 50 years and older to define older adults [16, 24, 25].

## Methods

The protocol for the systematic review, registered in PROSPERO under CRD42017056474 has been published elsewhere [26].

### Exclusion and inclusion criteria

Cross-sectional or follow-up studies published between 1 January 1980 and 28 May 2018 which reported a prevalence or incidence of hypertension in adults aged 50 years or above living in Africa were included. Original articles, conference proceedings or abstracts reporting the outcome of interest were also eligible for inclusion.

Hypertension was defined using the blood pressure 140/90 mmHg cut off or those taking anti-hypertensive treatment regardless of their blood pressure on measurement [27]. Where the definition was not explicitly provided and contact with the study authors to furnish this information had been unfruitful, we assumed that such studies, if published in or after 2010, had defined hypertension based on the 140/90 mmHg cut-off in conformity with the 7th Joint National Committee on Prevention, Detection, Evaluation, and Treatment of High Blood Pressure’s report of 2003 [27].

Articles were excluded if the older adults were pregnant, recruited from hospital patients or institutions for chronic or mental illness, or other restricted settings. They were also excluded when the subjects were African migrants, lived outside of Africa or the main outcome was self-reported hypertension or non-systemic hypertension. Review articles or expert opinion commentaries were excluded.

### Search strategy

We searched the major electronic databases, PubMed and Embase (via Ovid), as well as Academic Search Complete, CINAHL and PsycINFO (via EBSCOhost). In addition, we searched the African Journals Online repository and Google Scholar. The bibliography of the included studies was hand-search to identify additional studies. For grey literature, besides what was obtained via Ovid and Google Scholar, we searched OpenGrey and ProQuest.

The search terms were based on the population, intervention, comparator, outcome and settings (PICOS) approach modified to suit studies not involving interventions or comparison groups [28]. The terms for the intervention concept were “prevalence”, “proportion”, “survey”, “descriptive”, “cross-sectional”, “cohort”, “longitudinal”, "attributable fraction", and “incidence” (S1 Table). Those for the outcome were “hypertension”, "blood pressure", “cardiovascular” and “cardiometabolic”. Those for the settings were “Africa” and the names of 58 African countries and islands. Within each concept, the terms were linked with the “OR” operator” while the results combined across the concepts with the “AND” operator. We limited the search results to human studies conducted in the middle-aged (45 to 64 years)" or the "aged (65 and over)". Where, this was not possible, we used the terms "older adults", “elderly”, “middle-aged”, “geriatric”, “the aged”, and “senior”.

The search results from the different databases were managed using the Covidence software [29]. This programme automatically identified and removed duplicate papers. Then, the titles and abstracts were screened guided by the inclusion and exclusion criteria. The screening was facilitated by the use of key words such as “patients”, “African Americans” and “pregnant” which quickly enabled non-eligible studies to be reviewed and excluded. The full-text of potentially analyzable studies were retrieved and evaluated independently by two authors (WKB, JMKA). Discrepancies were resolved by consensus between the two reviewers, without recourse to an arbitrator. The Covidence software automatically generated the Preferred Reporting Items for Systematic Reviews and Meta-Analysis (PRISMA) flow chart of selected studies [28].

Where multiple publications existed on the same sample of subjects, we counted all such studies as one unique study. With these multiple papers, it was quite easy to identify the paper that most adequately provided the data for this review. Where necessary, complementary data were obtained from the additional reports. If hypertension was estimated in the same cohort at different time points, then these were reckoned as separate studies. Similarly, where a study covered more than one African country, then it was counted as one study that had provided multiple data points (reflecting the number of countries). There was no restriction on language of publication. Study authors were contacted to clarify or provide additional information on participants’ age, definition of hypertension employed or on the prevalence in specific groups as necessary.

### Data extraction

Data were extracted onto a preformatted previously piloted form in Excel. They included the study’s primary author, year of publication, setting, socio-demographics, objective, study design, study population, sampling procedure and size, participation rate, anthropometry, dietary and behavioural lifestyles, method of BP measurement, and the prevalence of hypertension and its grades where available [26]. The presence of co-morbidities such as diabetes or chronic kidney disease was recorded. Body mass index (BMI) cut off points of <18.5 kg/m^2^ defined underweight, 18.5–24.9 kg/m^2^ was normal weight, 25.0–29.9 kg/m^2^ was overweight and ≥30.0 kg/m^2^ was obese [20].

### Evaluation of study quality

The quality of the included studies was evaluated by two authors (WKB, JMKA) using the Hoy et al [30] tool which has been specifically validated for cross-sectional studies. The tool assesses external validity based on factors such as the representativeness of the sample, participation rate, and sampling methods. The internal validity is based on factors such as direct data collection from subjects or from proxy, suitability of case definition, reliability of study instrument, application of same measurement methods for all subjects and appropriateness of exposure period for the development or presence of hypertension in the subjects. In line with best practice, we avoided scoring the criteria [31] and instead, classified each study’s risk of bias as low, moderate and high based on an overall judgement on our confidence in the prevalence estimate after applying the criteria [30].

### GRADE assessment of the overall quality of evidence

The overall confidence in the estimates from the meta-analysis was assessed using the Grading of Recommendations Assessment, Development and Evaluation (GRADE), adapting the principles for rating systematic review of prognostic studies [32]. The overall level of evidence was rated as “high” where there is a high level of confidence that the pooled estimate lies close to the true population estimate or as “very low” where the confidence was very low. The rating categories in between were “moderate” and “low”. Unlike systematic reviews for treatment studies, the initial level of confidence in the estimates from longitudinal prognostic studies is rated as high [32]. Our initial level of confidence in the estimates from the prevalence studies was rated as moderate.

The five domains GRADE—risk of bias, imprecision, inconsistency, indirectness, and publication bias were considered in downgrading confidence in the overall estimate while the criteria of large effect, dose response gradient, and direction of plausible confounding were considered in upgrading the confidence in the estimates.

### Data analysis

The overall pooled prevalence across studies was estimated using a random effects model which involved first stabilizing the variances of the prevalence estimates through Freeman-Tukey arcsine transformation [33]. Heterogeneity between the included studies was assessed with the chi-squared test on Cochran’s Q (alpha set at 0.1) statistic [34] and the Higgins and Thompson's I^2^ statistic [35]. Separate sub-group analyses were performed based on sex, age group, urban-rural residence, sub-region, study design and year of publication grouped in 5-year periods. To further explore heterogeneity, we performed a post-hoc random-effects meta-regression analysis using the residual restricted maximum likelihood method [36]. We included five continuous covariates (study year, publication year sample size enrolled, percentage of obesity and percentage with no education in the total sample) and one categorical variable (automated versus manual type blood pressure monitor) individually in the model. In the combined model, we included four covariates which enabled a yield of ten or more observations for analysis.

We assessed the effect of excluding studies deemed to be at high risk of bias on the pooled estimate. We performed an influence analysis by computing the summary estimate of the prevalence of hypertension after removing one included study at a time. Forest plots provided a visual image of the point and 95% interval prevalence estimate as well as the summary estimate. Statistical analyses were performed in Stata version 14 [37].

The presence of reporting bias was determined through funnel plot asymmetry, Duval and Tweedie’s trim and fill technique [38] and Egger’s test [39]. Statistical significance was fixed at p value <0.05. The reporting of this review conforms to the PRISMA 2009 guidelines (S2 Table).

## Results

### Study flow and characteristics

**Fig 1:**
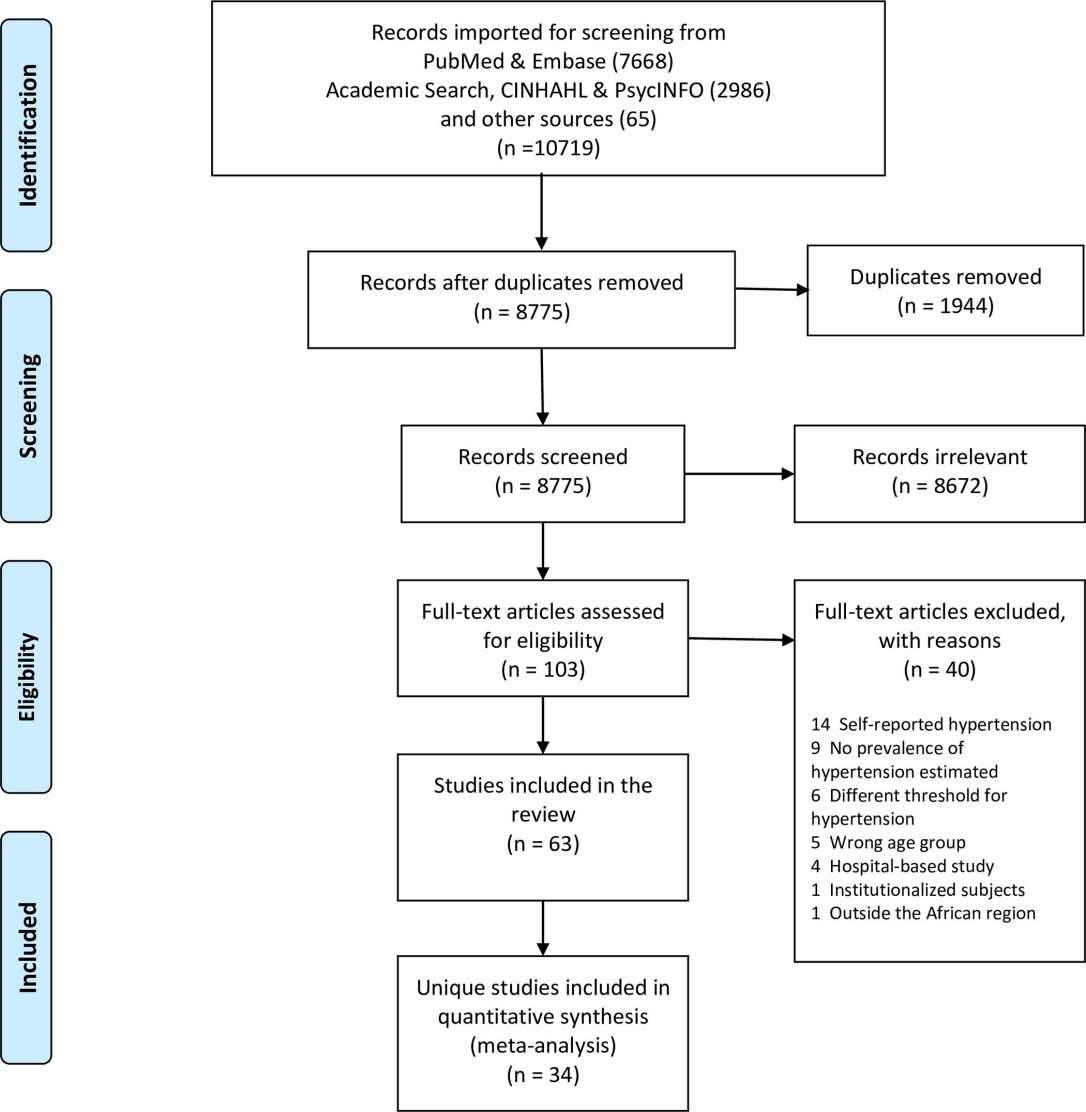
PRISMA flow diagram showing study selection.

The literature search including the hand-search yielded 10,719 articles, out of which 1,944 duplicate studies were excluded (Fig 1). Following the title and abstract review, 103 full-text articles were retrieved and evaluated for inclusion. Forty of these full-text articles were excluded for various reasons such as using self-reported estimate of hypertension or not estimating the prevalence of hypertension at all (Fig 1, S3 Table). Of the remaining 63 eligible full text articles, 34 unique studies published from 2005 to 2018 were retained for analysis, after taking into account, multiple studies on the same study subjects (S4 Table). These studies, three of which covered multiple countries or cohorts, provided a total of 37 data contributions (Table 1). Four (11.7%) studies were published in 2005–2009, 18 (52.9%) in 2010–2014 and 12 (35.3%) in 2015–2018 (Table 1). The earliest reported start year of the data collection was October 2000 [40] while the latest reported end date was 2016 [65]. The duration of data collection ranged from 2.5 months [69] to 24 months [58].

**Table 1:** Demographic characteristics of study sample and prevalence of hypertension in individual studies.

Primary Reference	Data collection period	Sub-region in Africa	Country	Location	Type of residence	Type of community-based participants	Enrolled sample size sex			Response rate		Age in years			Prevalence ≥140/90 mmHg		
							Men	Women	Total	Enrolment	BP measured	Range	% aged 70+ years	Mean age ± sd	Men	Women	Total
**Abegunde 2013** [41]		Western	Nigeria	Oyo State	Mixed	Adults aged ≥60 years	245	385	630	98.4	95.2	60–110	57.9	72.2 ± 9.5 (urban), 70.8 ± 8.1 (rural)	NR	NR	36.5
**Chami 2015** [42]	15 Jan 2012–15 Jun 2012	Northern	Algeria	Municipality of Sidi Bel-Abbes	Urban	Adults aged ≥65 years with diabetes	159	234	393	92.5	100.0	NR	NR	75.9 ± 7.1	NR	NR	78.1
**Dewhurst 2013** [43]	1 Nov 2009–31 Jul 2010	Eastern	Tanzania	Hai district	Rural	Adults aged ≥70 years	972	1251	2223	93.5	99.6	NR	100.0	NR	62.2	75.8	69.9
**Duboz 2015** [44]	Ferlo, Aug 2010; Dakar 2008	Western	Senegal	Ferlo village; Dakar city	Mixed	Adults aged ≥50 years	285	413	698	NR	100.0	NR	30.2	Dakar 65.37 ± 9.93; Ferlo 62.22 ± 8.73	NR	NR	59.2
**El Tallawy 2012** [45]	1 Apr 2005–31 Jul 2005	Northern	Egypt	Al Kharga district		Adults aged ≥50 years	NR	NR	219	>99%	74.9	NR	NR	NR	NR	NR	31.1
**Fajemilehin 2005** [46]		Western	Nigeria	Ife Central, Ilesa West and Obokun LGAs in Osun State	Mixed	Adults ≥60 years	NR	NR	681		97.2	NR	NR	NR	NR	NR	68.1
**Gray 2016** [47]	Jan 2013—May 2013	Eastern	Tanzania	Hai district	Rural	Elderly people aged ≥70 years	835	1038	1873	83.9	99.6	NR	100.0	NR	NR	NR	73.2
**Guerchet 2012A** [48]	Sept 2008—Mar 2009	Central	CAR	Bangui	Urban	Adults aged ≥65 years	NR	NR	496	97.4	99.6	NR	NR	NR	NR	NR	51.8
**Guerchet 2012B** [48]	Sept 2008—Mar 2009	Central	Congo	Brazzaville	Urban	Adults aged ≥65 years	NR	NR	520	95.2	99.8	NR	NR	NR	NR	NR	55.7
**Hammami 2011** [49]	Dec 2008—Feb 2009	Northern	Tunisia	Centre-ouest	Urban	Adults aged ≥65 years	202	396	598	100.0	100.0	65–95	62.0	72.3 ± 7.4	45.0	55.6	52.0
**Hien 2014** [50]	Aug 2012- Dec 2012	Western	Burkina Faso	Bobo-Dioulasso	Urban	Elderly aged ≥60 years	215	174	389	100.0	100.0	NR	39.3	69 ± 7	79.1	86.2	82.3
**Ice 2008** [51]	2005	Eastern	Kenya	Nyando, and Kisumu Rural districts in Nyanza province	Rural	Grandparent caregivers and non-caregivers aged >60 years	138	149	287	NR	100.0	NR	NR	72.6 ± 7.6	NR	NR	24.4
**Iribhogbe 2013** [52]	Aug 2012—Oct 2012	Western	Nigeria	Ekpoma Esan West Local Government Area of Edo State	Urban	Church attendants aged ≥50 years	100	100	200	100.0	100.0	NR	23.5	67.55 ± 16.61	58.0	35.0	46.5
**Ivy 2015** [53]	6 March 2013–1 June 2013	Eastern	Tanzania	Hai district	Rural	Adults aged ≥70 years in 2 villages	42	37	79	95.2	100.0	NR	100.0	median 76y	NR	NR	78.5
**Kamoun 2006** [40]	Oct 2000—Sept 2001	Northern	Tunisia	National	Mixed	Adults aged ≥65 years	487	494	981	98.2	100.0	65–104	65.3	73.1 ± 6.8	NR	NR	69.8
**Kinyanda 2016** [54]	June 2009—Apr 2010	Eastern	Uganda	Rural area of Masaka district, and peri-urban Wakiso district	Mixed	Adults aged ≥50 years participating in the Wellbeing of Older People’s Cohort Study	176	292	468	99.4	100.0	NR	31.8	NR	NR	NR	37.2
**Koopman 2012** [55]	2009	Western	Ghana	Garu-Tempane District	Rural	Registered adults aged ≥50 years	480	444	924	85.4	100.0	NR	35.3	NR	25.6	22.5	24.1
**Lasisi 2010** [56]	2008	Western	Nigeria	8 contiguous Yoruba-speaking states in the Nigeria	Mixed	Adults aged ≥65 years	NR	NR	1474	78.7	12.5	NR	92.2	NR	NR	NR	22.3
**Macia 2012** [57]	Jan 2009—Jun 2009	Western	Senegal	Dakar	Urban	Adults aged ≥50 years	263	237	500	NR	100.0	NR	19.2	NR	63.9	67.1	65.4
**Mathenge 2010** [58]	Jan 2007—Dec 2008	Eastern	Kenya	Nakurua district	Mixed	Adults aged ≥50 years	2095	2301	4396	87.7	100.0	50–80+	26.4	NR	NR	NR	50.1
**Minicuci 2014** [59]	Jan 2007—Dec 2008	Western	Ghana	National	Mixed	Adults aged ≥50 years	2348	2376	4724	95.9	100.0	NR	32.5	NR	51.5	50.7	51.1
**Mkhize 2013** [60]	2008	Northern	South Africa	Umlazi, Kwazulu Natal		Registered pensioners aged ≥60 years	46	224	270	NR	100.0	NR	NR	NR	NR	NR	90.0
**Mugisha 2013** [61]	Jan 2012—Jan 2013	Eastern	Uganda	Kalungu district	Rural	Adults aged ≥50 years	605	844	1449	72.3	100.0	NR	27.7	median 62	36.5	38.3	37.5
**Nuertey 2017** [62]	Apr 2014—Dec 2014	Western	Ghana	National	Mixed	Registered pensioners who presented for medical screening	3069	1402	4471	93.0	99.2	Min 56	28.5	67.2 ± 5.4	NR	NR	47.8
**Ochayi 2006** [63]	Jan 2002—Dec 2002	Western	Nigeria	Jos South Local Government Area	Mixed	Adults aged ≥65 years	136	144	280	94.9	100.0	NR	NR	73.0 ± 9.0	NR	NR	39.6
**Ogunniyi 2011A** [64]	2001–2007	Western	Nigeria	Elderly Yoruba residents 65+ years in Idikan Ward of Ibadan City, Nigeria	Mixed	Non-demented subjects aged ≥65 years from a 2001 baseline cohort	892	1826	2718	Number eligible in 2001 not reported	99.5	NR	NR	76.18 ± 5.35; 77.51 ± 6.03	NR	NR	62.1
**Ogunniyi 2011B** [64]	2001–2007	Western	Nigeria	Elderly Yoruba residents 65+ years in Idikan Ward of Ibadan City, Nigeria	Mixed	Non-demented subjects aged ≥65 years from the 2001 evaluation wave who were re-evaluated during the 2004 and 2007 waves for dementia	543	1210	1753	64.5	64.5	NR	NR	NR	NR	NR	60.9
**Osman 2017** [65]	Nov 2016—Dec 2016	Western	Ghana	4 districts in Ashanti Region	Mixed	Adults aged ≥65 years	135	265	400	100.0	100.0	NR	NR	NR	57.8	52.5	54.3
**Paddick 2015** [66]	Apr 2010—Mar 2014	Eastern	Tanzania	Hai district	Rural	Adults aged ≥70 years with mild cognitive impairment	10	36	46	15.5	15.5	NR	100.0	NR	NR	NR	69.6
**Peltzer 2013** [67]	2007	Southern	South Africa	National	Mixed	Adults aged ≥ 50 years	1638	2202	3840	60.0	95.6	NR	23.8		74.4	79.6	77.4
**Pilleron 2017A** [68]	Nov 2011—Dec 2012	Central	CAR	Bangui, Nola	Mixed	Adults aged ≥65 years	366	601	967	94.7	100.0	65–93	60.4	72.8 ± 6.5, 72.6 ± 6.4	NR	NR	53.7
**Pilleron 2017B** [68]	Nov 2011—Dec 2012	Central	Congo	Brazzaville, Gamboma	Mixed	Adults aged ≥65 years	400	623	1023	94.7	100.0	65–99	66.8	74.0 ± 6.9; 73.6 ± 6.7	NR	NR	68.0
**Putnam 2018** [69]	29 Feb 2016–13 May 2016	Eastern	Tanzania	Hai district	Rural	Sub-sample of a cohort aged ≥70 years who were seen at baseline in 2009/10, and also in 2012/13	116	130	246	59.7	100.0	NR	100.0	NR	NR	NR	67.5
**Raji 2017** [70]	Baseline cohort of 2003/2004 followed up in Wave 1 in 2007	Western	Nigeria	8 contiguous states in the Southwestern and North central regions	Mixed	Adults aged ≥ 65 years, part of Ibadan Study of Ageing	660	809	1469	76.8	100.0	NR	79.0	76.9 ± 8.4	62.0	70.7	66.8
**Scholten 2011** [71]	Jun 2009—Apr 2010	Eastern	Uganda	Rural area of Masaka district, and peri-urban Wakiso district	Mixed	Adults aged ≥50 years	198	312	510	>99%	100.0	NR	34.9	NR	30.3	34.6	32.9
**Tianyi 2017** [72]	May 2013—July 2013	Central	Cameroon	Batibo health district	Rural	Adults aged ≥50 years	156	345	501		100.0	50–110	24.2	62.7 ± 9	60.9	55.7	57.3
**Yerly 2013** [73]	2004	Eastern	Seychelles	National	Mixed	Adults aged 50 to 64 years	151	178	329	83.7	93.2	50–64	0.0	NR	NR	NR	69.9

BP = blood pressure; CAR = Central African Republic; LGA = Local Government Area; NR = not reported, sd = standard deviation

Two sets of studies covered two different countries (Central African Republic and Congo) were analyzed separately for each country [48, 68]. One longitudinal study in Nigeria covering two different time periods, six years apart, provided two data points [64]. Multiple publications reporting the prevalence of hypertension on the same study sample was observed in 19 (51.4%) studies. The most prolific involved nine papers published from the Study on global AGEing and adult health (SAGE) in South Africa in which the dataset was publicly available (S4 Table) [67].

The included studies were all original articles except for one dissertation [65]. Thirty-one data contributions (83.8%) were cross-sectional in design and six were follow-up studies. Eleven (29.7%) involved demographic surveillance sites or a population cohort. In half of the 34 studies, hypertension was part of the main objective of the study.

Thirty-one (91.2%) studies were published in English and three (8.8%) in French. Only five (13.5%) data contributions were from national studies (Table 1). The included data contributions were conducted in 15 countries—eight in Nigeria, five in Tanzania, four studies in Ghana, three in Uganda, two each in Central African Republic (CAR), Congo, Kenya, Senegal, South Africa and

Tunisia and one each in Algeria, Burkina Faso, Cameroon, Egypt and Seychelles. Thus, there were two (5.4%) data contributions from southern Africa, four (10.8%) from northern Africa, five (13.5%) from central Africa, 11 (29.7%) from eastern Africa and 15 (40.5%) from western Africa. Nine (24.3%) data contributions covered rural populations, eight (21.6%) covered urban populations while the remaining 20 (54.1%) covered mixed populations.

A total of 43,025 subjects and a median of 598 subjects were enrolled across the data contributions (Table 1). In the individual studies, the total sample size ranged from 46 to 4,724 participants. The lowest number of participants were observed in studies from the eastern Africa, in urban populations or conducted in the 2005–2009 period. Their ages ranged from 50 to 110 years [72]. The sex distribution of participants was provided by 32 studies which reported a median of 254 males and 391 females. In these studies, females constituted 54.2% of the total number of enrolled subjects. Of the total enrolled subjects, 95.6% (range 12.5% - 100%) had their blood pressures measured.

In 23 studies reporting educational attainment of the enrolled participants, the proportion who did not have any formal education ranged from 12.1% [62] to 93.8% [41]. In thirteen of these studies, more than half of participants did not have any education. Study participants were also widely diverse in their body build. In the 13 studies that reported this information, the proportion who were overweight or obese (body mass index ≥ 25 kg/m^2^) ranged from 0.8% [55] to 80.0% [49].

Two studies [45, 52], both published in 2013, did not explicitly provide the blood pressure cut-offs used to define hypertension. Contacts with the study authors failed to elicit this information. They were both deemed to have used the 140/90mmHg cut-off in line with the JNC 7.

### Blood pressure measurement

Of the 34 included studies, five [42, 44–46, 56] did not provide information on how blood pressure was measured. Studies that did, only provided scant information which showed differences in the protocol in terms of the number of visits at which blood pressure was measured, frequency of measurements per visit, the readings used in the analysis, the posture of the subject and the part of the body on which measurements were taken (Table 2).

**Table 2:** Blood pressure measurement methods used in the included studies on hypertension in older adults in Africa.

No.	Primary Reference	Personnel taking BP	No. of visits	Frequency of readings per visit	Initial rest time (mins)	Interval between multiple readings (mins)	Reading used in analysis	Cuff size	Posture of subject	Part of body on which BP taken	BP Device
1	Abegunde 2013 [41]	Principal investigator	1	2	≥5 mins		mean of 2 readings	appropriate cuff size	seated upright	right arm	manual mercury
2	Dewhurst 2013 [43]		1	3	5 mins	1 min	mean of last 2 readings, if within 10 mmHg of each other	appropriate cuff size	seated upright	right arm	electronic
3	Gray 2016 [47]		1	3	5 mins	1 min	mean of last 2 readings, if within 10 mmHg (SBP or DBP) of each other	appropriate cuff size	seated upright	right arm	electronic
4	Guerchet 2012 [48]		1	4		5 mins	mean of four measurements, twice on each arm		lying supine	each arm	
5	Hammami 2011 [49]	3 trained doctors	1	2	5 mins		mean of 2 readings		seated upright	not reported	unspecified
6	Hien 2014 [50]	2 doctors	1	3	≥5 mins		mean of last 2 readings		seated upright	left arm	electronic
7	Ice 2008 [51]	a local clinical officer and an osteopathic medical student assistant	1	3	≥5 mins	not specified	mean of 3 readings	a standard cuff	seated upright	right arm	manual unspecified type
8	Iribhogbe 2013 [52]	a team of doctors	1							not reported	
9	Ivy 2015 [53]	Census enumerators supervised by medical students	1	3	5 mins	1 min	mean of last 2 readings, if within 20 mmHg (SBP) or 10 mmHg (DBP) of each other	appropriate cuff size	seated upright	right arm	electronic
10	Kamoun 2006 [40]		1		20–30 mins				seated upright	not reported	
11	Kinyanda 2016 [54]		1	3			mean of 3 readings			not reported	
12	Koopman 2012 [55]		1	1					lying supine	any arm	manual unspecified type
13	Macia 2012 [57]	4 PhD students in the departments of Medicine and Pharmacy	1	2		15–20 mins	mean of 2 readings		seated upright		electronic
14	Mathenge 2010 [58]	a nurse	1	3	5 mins	≥5 mins	mean of last 2 readings	medium cuff size was used to fit arms 22 to 32 cm	seated upright	right arm	electronic
15	Minicuci 2014 [59]		1	3		1 min	mean of last 2 readings			wrist	electronic
16	Mkhize 2013 [60]	registered nurse	1	2			mean of 2 readings			not reported	unspecified
17	Mugisha 2013 [61]	2 trained nurses and 3 non-medical interviewers	1	3	≥15 mins	3 mins	mean of last 2 readings	appropriate cuff size	seated upright	unspecified arm	electronic
18	Nuertey 2017 [62]	doctors	1					appropriate cuff sizes		not reported	electronic
19	Ochayi 2006 [63]		1						seated upright	not reported	unspecified
20	Ogunniyi 2011 [64]	trained interviewers	1	3	20 mins	15 mins	mean of 3 readings		seated upright	right arm	
21	Osman 2017 [65]		3 (daily for 3 days)	1			mean of 3 readings		seated upright	left arm	electronic
22	Paddick 2015 [66]	doctors and local nurse	1	3	5 mins	1 min	mean of last 2 readings, if within 10 mmHg (SBP or DBP) of each other		seated upright	right arm	electronic
23	Peltzer 2013 [67]	not reported	1	3			mean of last 2 readings		seated upright	right arm or wrist	electronic
24	Pilleron 2017 [68]	medical residents and nurses	1	4		≥5 mins	mean of four measurements, twice on each arm		lying supine	each arm	manual mercury
25	Putnam 2018 [69]	medical residents and enumerators	1	3	5 mins	1 min	mean of last 2 readings, if within 20 mmHg (SBP) or 10 mmHg (DBP) of each other	appropriate cuff size	seated upright	right arm	electronic
26	Raji 2017 [70]		1	3	≥5 mins	5 mins	mean of 3 readings		seated upright	not reported	electronic
27	Scholten 2011 [71]	trained interviewers	1	3			median value of 3 readings		seated upright	wrist	electronic
28	Tianyi 2017 [72]	not reported	1	2	≥10 mins	≥5 mins	mean of 2 readings	cuff covered at least 80% of arm	seated upright	unspecified arm	manual unspecified type
29	Yerly 2013 [73]		1	3	≥30 mins	≥2 mins	mean of last 2 readings		seated upright	not reported	manual mercury

BP = blood pressure

For most studies, the initial rest period before measurements were taken ranged from 5 minutes to at least 30 minutes. All but one study measured the blood pressure at a single visit. The one study using multiple visits measured blood pressure for all subjects daily for three days along with interviews about food frequency [65]. The frequency of measurements at a visit ranged from one to four. Of the 23 studies reporting which blood pressure measurements were used in the analysis, 11 (47.8%) reported using the mean of the last two of three readings while one used the median of three readings. Five studies reported analysing the mean of the two recorded blood pressures.

Except for three studies which measured the blood pressure in the supine position, all the studies had their subjects seated upright. Blood pressures were mostly taken on the arm but were taken on the wrist in two studies [59, 71] or on either the either the arm or wrist in one study [67]. Majority of studies measured the blood pressure on right arm although three studies measured it on either arm [55] or on both arms [48, 68]. Electronic automated blood pressure monitors were mostly used although a few studies used the standard mercury sphygmomanometer. Those taking the blood pressure measurements also varied from well-trained professional doctors to non-medical workers.

### Prevalence of hypertension

**Fig 2:**
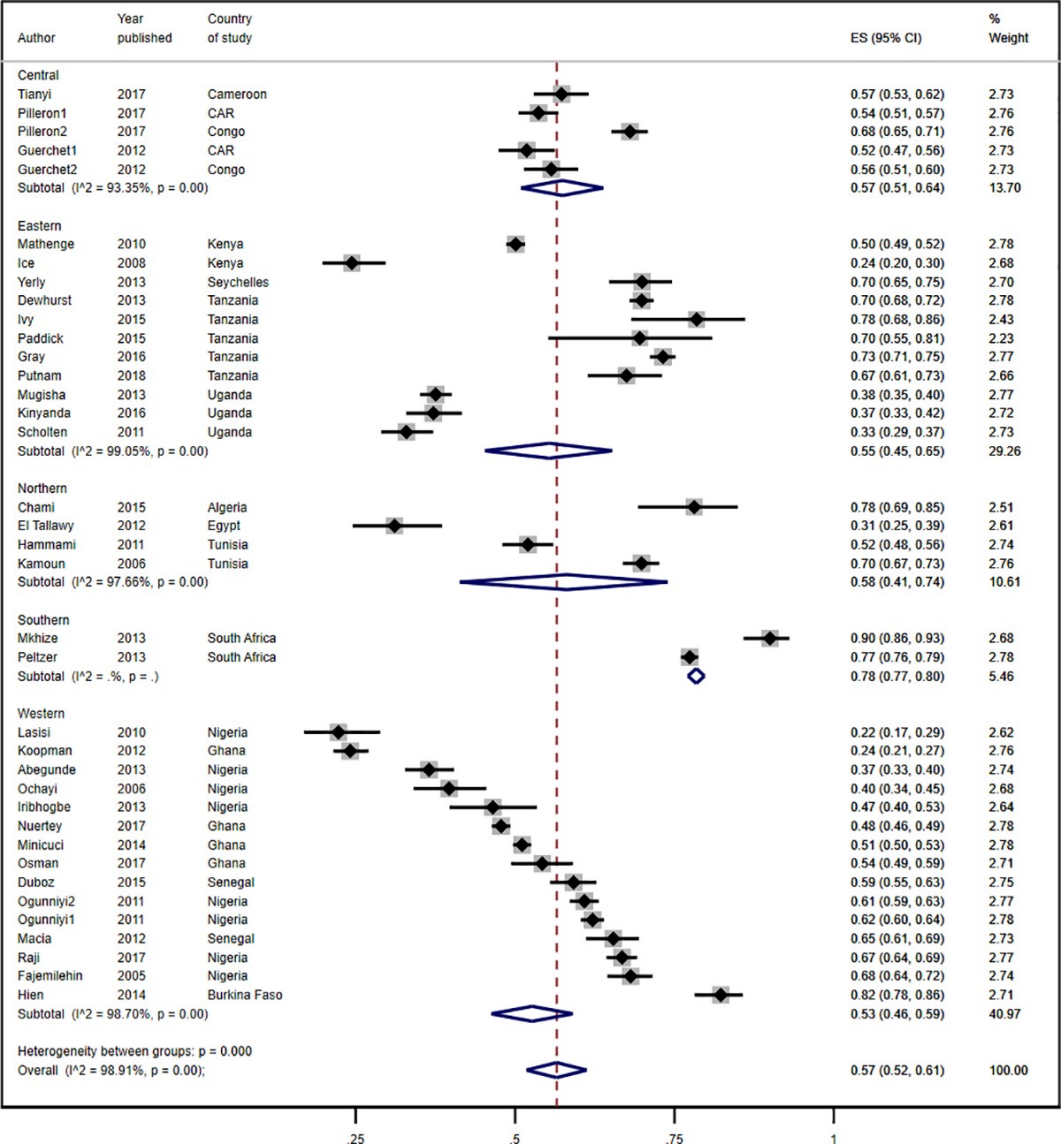
Prevalence estimates of hypertension by African sub-region.

The prevalence of hypertension ranged from 22.3% in community-based elderly residents in the Yoruba-speaking areas of Nigeria [56] to 90.0% among elderly residents in Umlazi township in South Africa [60, 74]. A combined total of 23,508 (57.2%) participants across the studies who had their blood pressure measured or who were on anti-hypertensive medication were reported to have hypertension. The overall pooled estimate across 37 data points from the random effects model was 57.0% (95% CI 52%-61%; Fig 2). It was not statistically significantly different between cross-sectional studies [56.0% (95% CI 51.0%-61.0%)] and follow-up studies [59.0% (51.0%-67.0%)].

**Fig 3:**
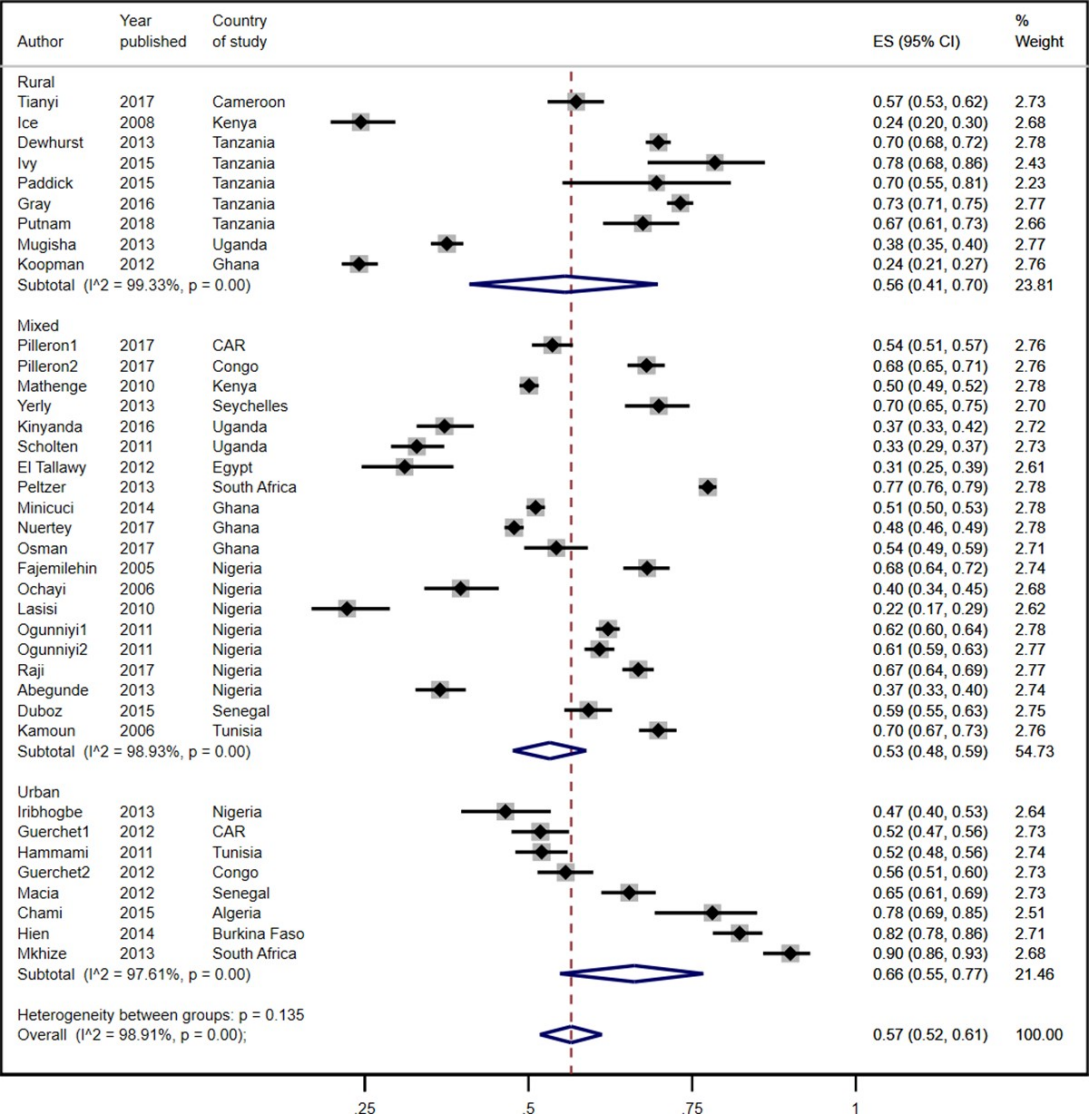
Prevalence estimates of hypertension by residence.

The pooled prevalence of hypertension of 78.0% (95% CI 77.0%-80.0%) in southern Africa was much higher than that in the other sub-regions of Africa where it ranged from 53% to 58% (Fig 2). The differences in the pooled prevalence of hypertension in the sub-regions other than southern Africa were not statistically significant. In terms of geolocality, the pooled prevalence of hypertension in urban residents was slightly higher than in rural populations but the difference was not statistically significant [57.0% (95% CI 52.0%-61.0%) versus 56.0% (95% 41.0%-70.0%); Fig 3]. Five of the nine studies in rural populations involved subjects aged 70 years and older. In the studies involving residents from both rural and urban areas, the pooled prevalence was 53.0% (95% CI 48.0%-59.0%).

**Fig 4:**
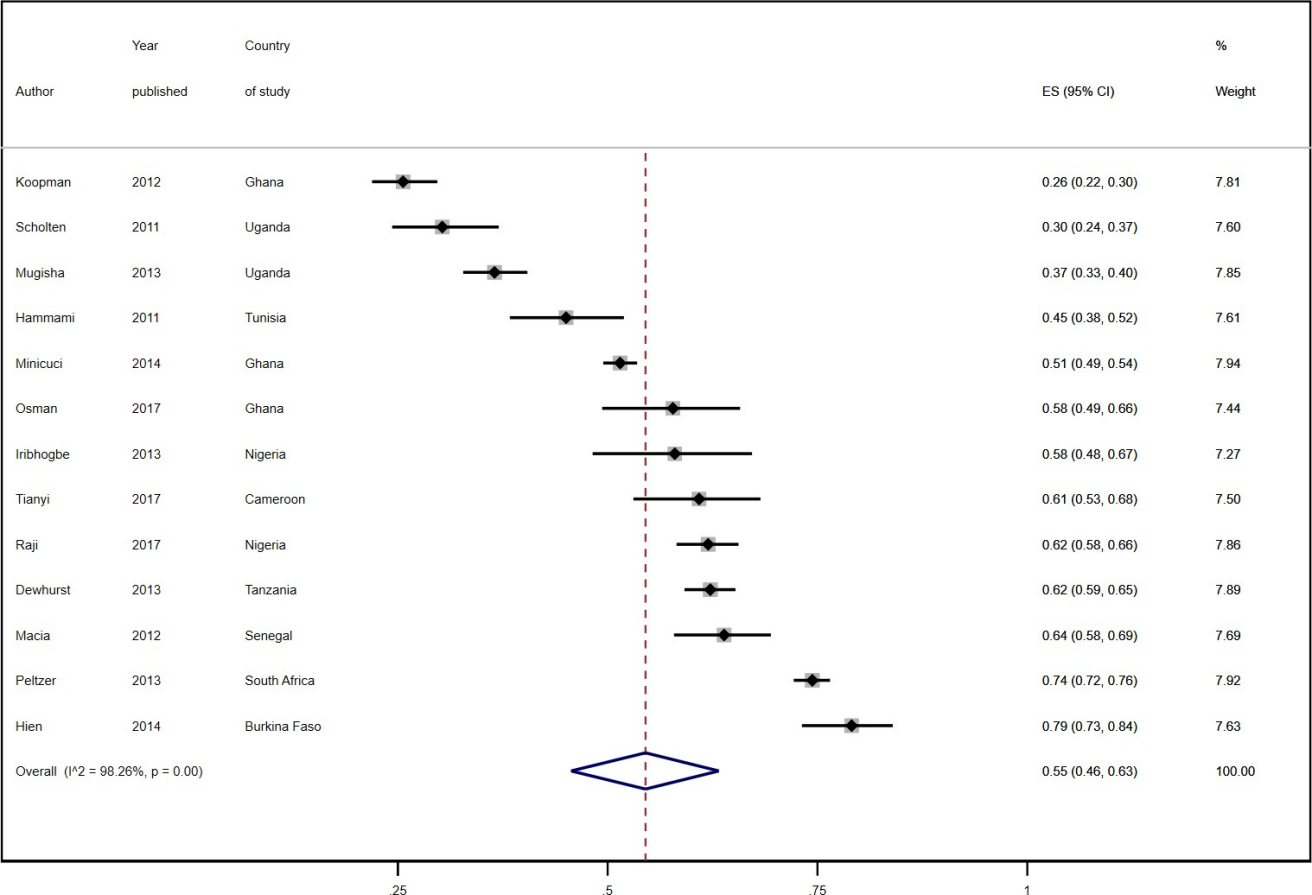
Prevalence estimates of hypertension in male subjects.

**Fig 5:**
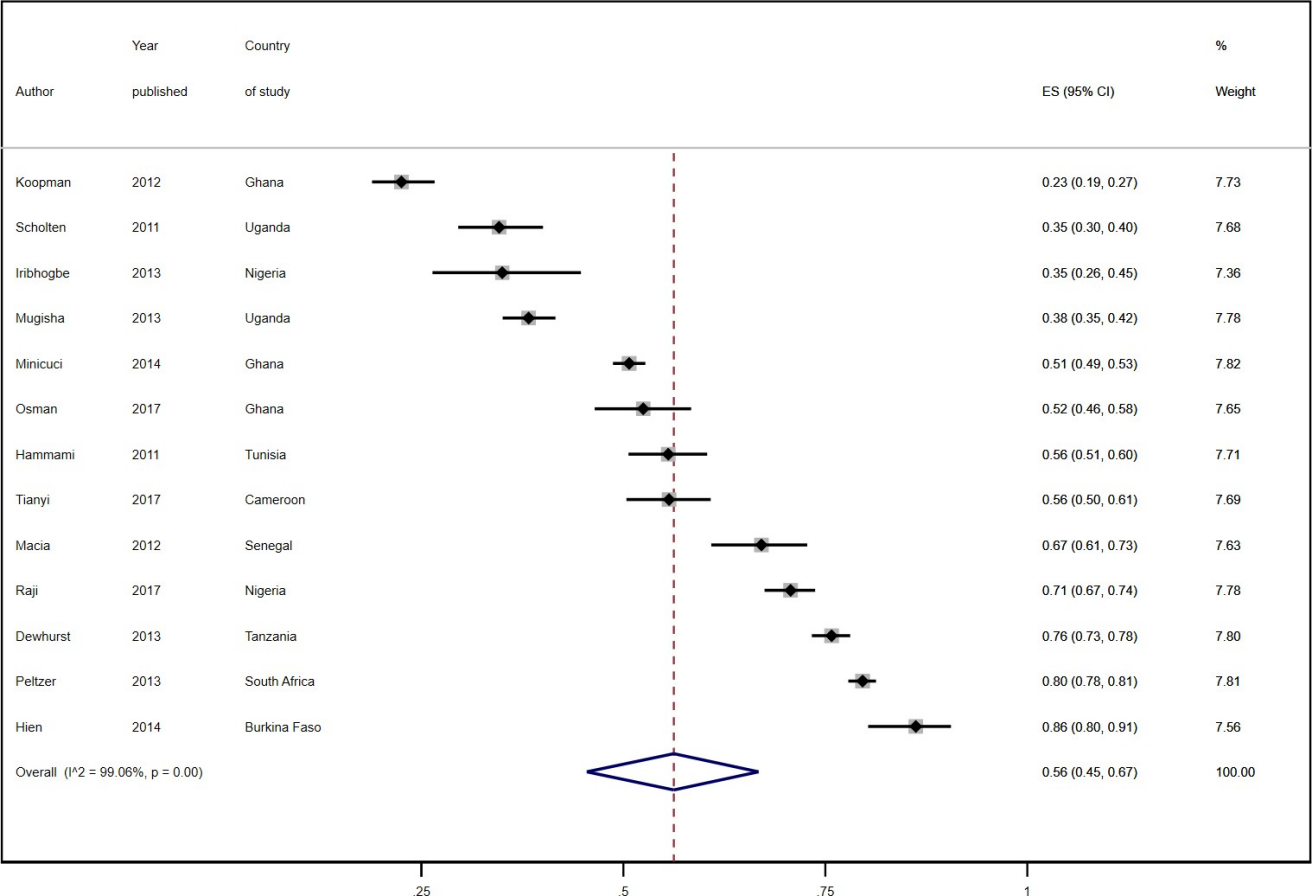
Prevalence estimates of hypertension in female subjects.

The overall pooled estimate from 13 studies reporting sex-specific prevalence of hypertension was 55.0% (95% CI 46.0%-63.0%) among males and 56.0% (95% CI 45.0%-67.0%) among females (Figs 4 and 5). In eight studies, the prevalence of hypertension was higher in females than in males [43, 49, 50, 57, 61, 67, 70, 71] while in the other five, it was higher in males (Table 1) [52, 55, 59, 65, 72].

The prevalence of hypertension increased with age. Twenty-six data contributions reported the age distribution of the older subjects (S1 Fig). In 14 of them, less than half of the subjects were aged 70 years and older. In the other twelve, this age group constituted at least half of the sample. The pooled prevalence of hypertension of the younger sample from the random effects model was 53.0% (95% CI 45.0%-61.0%) while that of the older sample as 61.0% (95% CI 54.0%-68.0%). In the third category in which the age distribution with respect to those aged 70 years and older was unknown, the summary prevalence was 57.0% (95% CI 52.0%-61.0%).

In the few individual studies reporting age-specific prevalence of hypertension, the prevalence was 3.5 to 13.5 percentage points higher in the oldest age group than in the youngest age group [43, 65, 68, 75]. The increase in the prevalence of hypertension with age was rarely monotonic [65] and tended to peak in the middle or penultimate age group [49, 68, 72, 75]. In all the studies in which the relationship between age and hypertension was investigated through multivariate analysis, older age group was independently associated with hypertension [43, 57, 68, 76, 77].

The prevalence of hypertension increased by the year group of publication, but the differences were not statistically significant. In the four data contributions published in 2005–2009, the prevalence ranged from 24.0% to 70.0% with a pooled estimate of 51.0% (95% CI 30.0%-71.0%). The overall pooled estimate for the 20 data points from studies published in 2010–2014 was 54.0% (95% CI 47.0%-61.0%) while that for the 13 data points from studies published in 2015–2019 was 62.0% (95% CI 55.0%-69.0%). However, the difference became statistically significantly when the studies were dichotomized into those published before the median publication year, 2013 (47.0%, 95% CI 40.0%-55.0%) versus those published at or after the median year (63.0%, 95% CI 57.0%-69.0%). In a cohort of elderly adults in the Ibadan Ageing Study in Nigeria, the prevalence of hypertension declined slightly from a baseline of 62.1% to 60.9% six years later [64].

The relationship between body mass index (BMI) and hypertension was reported by 13 studies. In multivariate analyses, older adults who were overweight or obese were up to 3.5 times as likely as those with normal BMI to have hypertension [72, 76, 77]. A dose-response relationship was observed in several studies [49, 78, 79].

### Evaluation of bias and sensitivity analysis

Using the Hoy tool [30], we determined that eight studies had a high risk of biased estimate [44, 46, 51, 52, 54, 62, 65, 66] and nine studies [40, 47, 53, 56, 57, 61, 67, 69, 70] had a moderate-risk of bias while the remaining 17 studies had low risk of bias (S5 Table). The pooled prevalence of hypertension did not differ by the study’s evaluated risk of bias (S2 Fig). The prevalence of hypertension in high and low risk biased studies was 50.0% (95% CI 42.0%-59.0%) and 56.0% (95% CI 50.0%-62.0%) respectively. Excluding the studies with high risk of bias did not have any significant impact of the pooled prevalence of hypertension.

In the influence analysis, the removal of a high prevalence (77.4%, 95% CI 76.0%-78.7%) study [67] resulted in a significant reduction in the pooled estimate from 57.0% (95% CI 52%-61%) to 55.9% (95% CI 55.4%-56.4%) (S3 Fig). Conversely, the omission of two studies, one at a time, with respective low prevalence of 24.1% (95% CI 21.4%-27.0%) [55] and 47.8% (95% CI 46.3%-49.3%) [62] resulted in a significant increase of the overall pooled prevalence to 59.3% (95% CI 58.9%-59.8%) and 59.5% (95% CI 59.0%-60.0%). Thus, while the removal of these three studies, in turn, resulted in a significant change in the summary estimate, the magnitude of the change was small—within 2.5 percentage points of the original summary estimate.

### Sources of heterogeneity

The I^2^ statistic measures the proportion of the total variability explained by heterogeneity while the Cochran's Q statistic tests whether all studies are evaluating the same effect. There was substantial heterogeneity across the included studies as indicated by the I^2^ statistic of 98.9% and the heterogeneity chi-square test (p <0.0001). The sub-group analysis based on sex, study design, type of residence or sub-region failed to reveal any source for the heterogeneity. The chi-squared statistical tests for subgroup differences consistently yielded p values <0.001.

In the univariate meta-regressions, the percentage of obesity in the total sample was the only statistically significant covariate, explaining 23.2% of the between-study heterogeneity in the prevalence of hypertension (S6 Table). The study year, publication year, sample size, percentage with no education and the type of blood pressure device did not explain any of the heterogeneity. However, in the combined model, the study year, publication year and the percentage obesity explained more of the heterogeneity (I^2^ = 87.5%, tau^2^ = 0.008 and adjusted R^2^ = 50.0%). The prevalence of hypertension increased with increasing year of publication and percentage obesity.

### Reporting bias

The presence of reporting bias was assessed by visually inspecting funnel plots for asymmetry. There was no evidence of asymmetry across all studies or by subgroups (S4 Fig). The Egger test failed to provide evidence for small-study effects on the prevalence (p value for bias = 0.711). Consistent with the Egger test, the ‘Duval and Tweedie’s trim and fill technique, which adjusts the pooled effect estimates to account for funnel plot asymmetry did not show evidence of reporting bias. The estimated prevalence from the random effects model between the observed and the adjusted values from filled model were the same (56.0%, 95% CI 51.4%-60.7%).

### GRADE assessment of quality of evidence

The data provided moderate quality evidence of the overall pooled prevalence although the study designs of the included studies were observational and so precluded blinding or randomization to reduce bias. We did not find any statistically significant differences in the pooled estimate by the level of bias in the studies. The influence analysis identified three studies with significant but small effect on the pooled estimate.

The evidence is however rated as very low on consistency due to the considerable heterogeneity and as moderate on precision due to the narrow confidence intervals engendered by the large sample size. Many studies recruited representative samples drawn from the general population and so we are moderately confident about their generalizability or applicability. On the publication bias domain, we have moderate confidence in the findings as we did not find sufficient evidence of publication bias from the funnel plots or Egger’s test.

## Discussion

We estimated that 57% of the adults aged ≥50 years in Africa had hypertension. This estimate is similar to the only other published review in which 55.2% of adults aged 55 years and older had hypertension [19]. In younger adults, the estimated prevalence is 30.0% to 30.8% [5, 6] in Africa or Sub-Saharan Africa [10].

As with other reviews in Africa, the pooled prevalence in our review was higher in urban than in rural populations but the difference was not statistically significant [6, 10, 80]. However, it is generally held that, a significantly greater proportion of urban populations are affected than rural populations [19]. The prevalence of hypertension in rural Africa appears to be much higher than previously thought and the urban-rural differences appear to be declining probably due to nutritional transition [5, 11, 12, 81–83].

Consistent with the recent review in older persons, we did not find statistically significant differences in the prevalence of hypertension by sub-region or sex [19]. Our findings are consistent with one review that found that the highest prevalence occurred in northern and southern Africa [5] but differ from those of another review that found that the lowest prevalence occurred in southern Africa [19]. The distribution of hypertension on the African continent may reflect that of body mass index, one of its major determinants [84–86].

The sex differences in hypertension have been variable. Consistent with our findings, other regional meta-analyses in Africa did not find significant sex differences [6, 10] while others reported higher male prevalence [5, 7]. In individual studies in older persons in Africa in which multivariable analyses were performed, female sex predicted a higher prevalence of hypertension [41, 43, 76, 77, 87] while in others sex was not independently associated with hypertension [49, 57, 67].

Very few systematic reviews and meta-analyses in Africa have assessed trends over time in the prevalence of hypertension. One review estimated an increase in the pooled prevalence from 19.7% in 1990 to 27.4% in 2000 and to 30.8% in 2010 [5]. Another study estimated an increase from 16.2% (95% CI 14.2–20.3) in 2008 to 17.4% (95% CI 15.4–22.6%) in 2025 [88]. More recently, the NCD Risk Factor Collaboration estimated that the age-standardized prevalence of raised blood pressure decreased globally but remained unchanged at around 34% in Sub-Saharan Africa in adults aged 18 years and older from 1975 to 2015 [89]. In Seychelles, the prevalence declined from 45% to 40% after a period of 15 years [90]. In the current review, the prevalence of hypertension increased by year of publication in the multivariate meta-regression model but not in the univariate model. More longitudinal studies are needed to better understand the trends in hypertension in Africa.

As expected, the prevalence of hypertension increased with BMI status in older people even after adjusting for potential confounders. A study involving institutional older adults in Tunisia also found that hypertension was 80% higher in obese subjects (86.9%) than in subjects with normal BMI [91]. Obesity is a well-recognized driver of hypertension in Africa with obese subjects being up to eight times as likely as those non-obese to have hypertension [86, 92, 93]. In view of the predominant association of overweight/obesity with hypertension and its increasing trend in Africa, there is an opportunity to explore the role of lifestyle interventions on improving the cardiovascular health of older adults [94, 95].

Overall, our review provided greater accuracy but confirmed most of the findings of the only other published review [19]. In particular, the pooled prevalence of hypertension was similar, and it did not vary significantly by age group, sex or sub-region. This was in spite of the fact that the target age group differed between the two reviews and there were only nine overlapping studies.

### Strengths and limitations

Our review adds to the scant knowledge on the prevalence of hypertension in older adults in Africa. To our knowledge, it is the first to focus on studies conducted exclusively among older adults aged 50 years and older in Africa. It is comprehensive in scope–covering multiple languages, study designs and article types. A major strength Is that the review captured studies in which the estimation of the prevalence was not the main objective of the study. It respects the PRISMA guidelines. We performed several quality assessments including the evaluation of the risk of bias in the included studies, performing sensitivity analysis, influence analysis and GRADE assessment of the meta-analysis.

Our review has some limitations. The major one is the substantial heterogeneity which undermine the pooling of the prevalence estimates and suggest that chance could be responsible for the between-study variability. Several other meta-analyses have reported similarly high levels of heterogeneity (*I*^*2*^ statistic 96%-99.5%) which may be due to the differences in the study participants, measurement methods and study design [6, 19, 96, 97]. We found that the participants included in our review differed widely in terms of their age distribution, proportion with no education and proportion who were obese. Moreover, their blood pressures were measured and captured for analysis using different methods. Sub-group analyses could not identify the sources of the heterogeneity. However, meta-regression analyses suggested that the obesity, year of publication and the study year explained some of the heterogeneity. Given the high heterogeneity, caution needs to be exercised in the interpretation of the findings. The cross-sectional design of most of the included studies is liable to such biases as selection and survival bias and explain moderate GRADE quality assessment of the meta-analysis. Future and on-going longitudinal studies in older adults may reduce some of these biases while allowing the trends in the prevalence of hypertension to be better measured.

It is not possible to impute causality between the explanatory variables and the main outcome in our review. Information on several variables was incomplete in some studies making comparison difficult. Given the low level of literacy among this older population, the date of birth of some participants was not always known and so their age was estimated using prominent historical local or national events [46, 63].

Two of the 34 papers did not explicitly provide information on the blood pressure threshold used to define hypertension. As the papers had been published recently, we assumed that they had used the 140/90mmHg cut-off. We do not think that this assumption had any impact on our findings. Support for our assumption was obtained in a paper published by the authors of one of the two studies in an adjoining governorate in Egypt in the same year in which their stated definition of hypertension was consistent with the above cut-off [98]. Moreover, the sensitivity analysis did not show that the findings from these papers had unduly affected the overall pooled estimate.

All but one of the included studies involved single visit blood pressure measurements, and so could overestimate the true prevalence of hypertension. One included study which used ambulatory blood pressure monitoring reported a 55.7% prevalence compared with 78.5% prevalence on the same subjects measured using conventional methods [53].

## Conclusions

In conclusion, we have estimated, after a comprehensive and meticulous study selection and analysis that, nearly three out of five older adults in rural and urban Africa have raised blood pressure. This review shows that the proportion has gradually increased between 2005 and 2018 and is high in the different sub-regions. Increasing obesity likely explains some of the increase in the prevalence of hypertension in older adults. Unfortunately, the older age group receives little priority in national and regional health programmes [99]. Given that, cardiovascular diseases can be prevented or managed in older adults, we call on health authorities to prioritize policies and commission and develop programmes to support the improvement of the cardiovascular health of older population in Africa while ensuring that younger adults stay healthy. Our findings suggest that weight reduction should be an important component of such programmes. These policies and programmes ought to be evaluated to see what works to promote active ageing. It is imperative that this is addressed within the framework of a life course approach and a national ageing programme, with support from the African Union, United Nations, Regional Economic Communities and the civil society. Older adults should be empowered and supported to organise themselves into active social and functional groups to serve as advocates and channels for improved health and social service delivery.

There is an urgent need for further research on hypertension in older adults on the continent. Longitudinal studies which use consistent study methods and blood pressure measurement protocol can fill in some of the data gaps including the trends and determinants of hypertension.

## Supporting information

S1 Table Search strategy for ovid medline and ovid embase databases. (DOCX)

S2 Table Preferred reporting items for systematic reviews and meta-analysis (PRISMA) 2009 checklist. (DOC)

S3 Table Full-text articles excluded after assessed for eligibility and reasons for exclusion. (DOCX)

S4 Table List of multiple studies on same subjects and their primary (unique) studies included in analysis. (DOCX)

S5 Table Quality assessment of included observational cohort and cross-sectional studies. (DOCX)

S6 Table Univariate and multivariate meta-regression exploring potential sources of heterogeneity in the prevalence of hypertension. (DOCX)

S1 Fig Prevalence estimates of hypertension by age group. (TIF)

S2 Fig Prevalence estimates of hypertension by risk of bias. (TIF)

S3 Fig Influence analysis omitting one study at a time from meta-analysis. (TIF)

S4 Fig Funnel plot for studies on the prevalence of hypertension. (TIF)

## References

[1] Institute for Health Metrics and Evaluation (IHME). Global Burden of Disease: GBD Compare Data Visualization Seattle, WA: IHME, University of Washington; 2018 [updated 28 December 2018; cited 2018]. Available from: https://vizhub.healthdata.org/gbd-compare/.

[2] O'Donnell MJ, Chin SL, Rangarajan S, Xavier D, Liu L, Zhang H, et al Global and regional effects of potentially modifiable risk factors associated with acute stroke in 32 countries (INTERSTROKE): a case-control study. Lancet. 2016;388(10046):761–75. 10.1016/S0140-6736(16)30506-2 27431356

[3] Livingston G, Sommerlad A, Orgeta V, Costafreda SG, Huntley J, Ames D, et al Dementia prevention, intervention, and care. 2017;390(10113):2673–734. 10.1016/S0140-6736(17)31363-6 28735855

[4] Akinyemi RO, Owolabi MO, Ihara M, Damasceno A, Ogunniyi A, Dotchin C, et al Stroke, cerebrovascular diseases and vascular cognitive impairment in Africa. Brain Res Bull. 2018 10.1016/j.brainresbull.2018.05.018 29807146 PMC6252289

[5] Adeloye D, Basquill C. Estimating the prevalence and awareness rates of hypertension in Africa: a systematic analysis. PLoS One. 2014;9(8):e104300 10.1371/journal.pone.0104300 25090232 PMC4121276

[6] Ataklte F, Erqou S, Kaptoge S, Taye B, Echouffo-Tcheugui JB, Kengne AP. Burden of undiagnosed hypertension in Sub-Saharan Africa: A systematic review and meta-analysis. Hypertension. 2015;65(2):291–8. 10.1161/HYPERTENSIONAHA.114.04394 25385758

[7] Bosu WK. The prevalence, awareness, and control of hypertension among workers in West Africa: a systematic review. Glob Health Action. 2015;8:26227 Epub 2015/01/28. 10.3402/gha.v8.26227 25623611 PMC4306751

[8] Addo J, Smeeth L, Leon DA. Hypertension in sub-saharan Africa: a systematic review. Hypertension. 2007;50(6):1012–8. Epub 2007/10/24. 10.1161/HYPERTENSIONAHA.107.093336 . 17954720

[9] Ndabarora E, Nishimwe C, Mukamusoni D. Systematic review of hypertension prevalence and awareness in Sub-Saharan Africa. Kibogora Polytechnic Scientific Journal. 2018;1:11–4.

[10] Sarki AM, Nduka CU, Stranges S, Kandala N-B, Uthman OA. Prevalence of Hypertension in Low-and Middle-Income Countries: A Systematic Review and Meta-Analysis. Medicine. 2015;94(50):e1959 10.1097/MD.0000000000001959 26683910 PMC5058882

[11] Addo J, Amoah AGB, Koram KA. The changing patterns of hypertension in Ghana: a study of four rural communities in the Ga district. Ethn Dis. 2006;16:894–99. 17061743

[12] Okeahialam BN, Ogbonna C, Otokwula AE, Joseph DE, Chuhwak EK, Isiguzoro IO. Cardiovascular epidemiological transition in a rural habitat of Nigeria: the case of Mangu Local Government Area. West Afr J Med. 2012;31(1):14–8. Epub 2012/11/02. . 23115090

[13] Awuah RB, Anarfi JK, Agyemang C, Ogedegbe G, Aikins Ad-G. Prevalence, awareness, treatment and control of hypertension in urban poor communities in Accra, Ghana. J Hypertens. 2014;32(6):1203–10. 10.1097/HJH.0000000000000165 24721931

[14] Etyang AO, Gerard Scott JAJGha. Medical causes of admissions to hospital among adults in Africa: a systematic review. Glob Health Action. 2013;6(19090). 10.3402/gha.v6i0.19090 23336616 PMC3541514

[15] Zhou B, Bentham J, Di Cesare M, Bixby H, Danaei G, Cowan MJ, et al Worldwide trends in blood pressure from 1975 to 2015: a pooled analysis of 1479 population-based measurement studies with 19.1 million participants. Lancet. 2017;389(10064):37–55. 10.1016/S0140-6736(16)31919-5 27863813 PMC5220163

[16] Duda RB, Anarfi JK, Adanu RM, Seffah J, Darko R, Hill AG. The health of the “older women” in Accra, Ghana: results of the Women’s Health Study of Accra. J Cross Cult Gerontol. 2011;26(3):299–314. 10.1007/s10823-011-9148-8 21695397

[17] Vasan RS, Beiser A, Seshadri S, Larson MG, Kannel WB, D'Agostino RB, et al Residual Lifetime Risk for Developing Hypertension in Middle-aged Women and Men: The Framingham Heart Study. JAMA. 2002;287(8):1003–10. 10.1001/jama.287.8.1003 11866648

[18] Aboderin I. Understanding and Advancing the Health of Older Populations in sub-Saharan Africa: Policy Perspectives and Evidence Needs. Public Health Rev. 2010;32(2):357–76. 10.1007/bf03391607

[19] Kaze AD, Schutte AE, Erqou S, Kengne AP, Echouffo-Tcheugui JB. Prevalence of hypertension in older people in Africa: a systematic review and meta-analysis. J Hypertens. 2017;35(7):1345–52. Epub 2017/03/08. 10.1097/HJH.0000000000001345 . 28267038

[20] WHO. WHO STEPS Surveillance Manual2008 16 May 2016 [cited 2016 16 May]. Available from: http://www.who.int/chp/steps/manual/en/.

[21] Apt NA. Aging in Africa: Past experiences and strategic directions. Ageing International. 2012;37(1):93–103.

[22] WHO. Global strategy and action plan on ageing and health (2016–2020). Geneva: World Health Organization, 2016.

[23] MDS Project Team. Proposed working definition of an older person in Africa for the Minimum Data Set project. Geneva: WHO, 2002.

[24] Aboyade OM, Beauclair R, Mbamalu ON, Puoane TR, Hughes GD. Health-seeking behaviours of older black women living with non-communicable diseases in an urban township in South Africa. BMC complementary and alternative medicine. 2016;16(1):410 Epub 2016/10/26. 10.1186/s12906-016-1378-4 27776505 PMC5078908

[25] Debpuur C, Welaga P, Wak G, Hodgson A. Self-reported health and functional limitations among older people in the Kassena-Nankana District, Ghana. Glob Health Action. 2010;3(Suppl 2):54–63. 10.3402/gha.v3i0.2151 PMC2957305 20963186

[26] Bosu WK, Aheto JMK, Zucchelli E, Reilly S. Prevalence, awareness, and associated risk factors of hypertension in older adults in Africa: a systematic review and meta-analysis protocol.(Report). Syst Rev. 2017;6(1). 10.1186/s13643-017-0585-5 28978358 PMC5628476

[27] Chobanian AV, Bakris GL, Black HR, Cushman WC, Green LA, Izzo JJL, et al The Seventh Report of the Joint National Committee on Prevention, Detection, Evaluation, and Treatment of High Blood Pressure: The JNC 7 Report. JAMA. 2003;289(19):2560–71. 10.1001/jama.289.19.2560 12748199

[28] Liberati A, Altman DG, Tetzlaff J, Mulrow C, Gøtzsche PC, Ioannidis JP, et al The PRISMA statement for reporting systematic reviews and meta-analyses of studies that evaluate health care interventions: explanation and elaboration. PLoS Med. 2009;6(7):e1000100 10.1371/journal.pmed.1000100 19621070 PMC2707010

[29] Veritas Health Innovation. Covidence systematic review software. Melbourne, Australia2018 [cited 2018]. Available from: www.covidence.org.

[30] Hoy D, Brooks P, Woolf A, Blyth F, March L, Bain C, et al Assessing risk of bias in prevalence studies: modification of an existing tool and evidence of interrater agreement. J Clin Epidemiol. 2012;65(9):934–9. 10.1016/j.jclinepi.2011.11.014 22742910

[31] Shamseer L, Moher D, Clarke M, Ghersi D, Liberati A, Petticrew M, et al Preferred reporting items for systematic review and meta-analysis protocols (PRISMA-P) 2015: elaboration and explanation. BMJ (Clinical research ed). 2015;349:g7647 10.1136/bmj.g7647 25555855

[32] Iorio A, Spencer FA, Falavigna M, Alba C, Lang E, Burnand B, et al Use of GRADE for assessment of evidence about prognosis: rating confidence in estimates of event rates in broad categories of patients. BMJ (Clinical research ed). 2015;350:h870 10.1136/bmj.h870. 25775931

[33] Nyaga VN, Arbyn M, Aerts M. Metaprop: a Stata command to perform meta-analysis of binomial data. Arch Public Health. 2014;72(1):39 10.1186/2049-3258-72-39 25810908 PMC4373114

[34] Cochran WG. The combination of estimates from different experiments. Biometrics. 1954;10(1):101–29.

[35] Higgins JP, Thompson SG, Deeks JJ, Altman DG. Measuring inconsistency in meta-analyses. BMJ. 2003;327(7414):557–60. Epub 2003/09/06. 10.1136/bmj.327.7414.557 12958120 PMC192859

[36] Harbord RM, Higgins JP. Meta-regression in Stata. The Stata Journal. 2008;8(4):493–519.

[37] StataCorp. Stata Statistical Software: Release 14.: College Station, TX: StataCorp LP; 2015.

[38] Duval S, Tweedie R. Trim and Fill: A Simple Funnel‐Plot–Based Method of Testing and Adjusting for Publication Bias in Meta‐Analysis. Biometrics. 2000;56(2):455–63. 10.1111/j.0006-341X.2000.00455.x 10877304

[39] Egger M, Davey Smith G, Schneider M, Minder C. Bias in meta-analysis detected by a simple, graphical test. BMJ. 1997;315(7109):629–34. Epub 1997/10/06. 9310563 10.1136/bmj.315.7109.629 PMC2127453

[40] Kamoun M, Hajem S, Gueddana N, Achour N, Slimane H. Diabetes, hypertension, obesity in elderly: Study about 981 Tunisian people aged 65 years and over. Revue Geriatr. 2006;31(7):477–86.

[41] Abegunde KA, Owoaje ET. Health problems and associated risk factors in selected urban and rural elderly population groups of South-West Nigeria. Ann Afr Med. 2013;12(2):90–7. 10.4103/1596-3519.112398 . 23713015

[42] Chami MA, Zemmour L, Midoun N, Belhadj M. Diabetes mellitus in the elderly: The first Algerian survey. Medecine des Maladies Metaboliques. 2015;9(2):210–5. 10.1016/S1957-2557(15)30046-8.

[43] Dewhurst MJ, Dewhurst F, Gray WK, Chaote P, Orega GP, Walker RW. The high prevalence of hypertension in rural-dwelling Tanzanian older adults and the disparity between detection, treatment and control: a rule of sixths? J Hum Hypertens. 2013;27(6):374–80. 10.1038/jhh.2012.59 . 23235367

[44] Duboz P, Touré M, Hane F, Macia E, Coume M, Bâ A, et al Vieillissement et pathologies chroniques au Sénégal. Comparaison entre des populations vivant en milieu rural (Ferlo) et urbain (Dakar) Ageing and chronic diseases in Senegal. A comparison between rural (Ferlo) and urban (Dakar) populations. Bull Soc Pathol Exot. 2015;108(1):25–31. 10.1007/s13149-014-0397-y 25256252

[45] El Tallawy HN, Farghly WM, Shehata GA, Rageh TA, Hakeem NA, Abo-Elfetoh N, et al Prevalence of dementia in Al Kharga District, New Valley Governorate, Egypt. Neuroepidemiology. 2012;38(3):130–7. Epub 2012/03/22. 10.1159/000335655 . 22433971

[46] Fajemilehin BR, Ojfeitimi EO, Adegbehingbe BO, Asa SS, Bamiwuye SG, Owolabi OO. Nutritional and health status in an elderly population in Nigeria. Afr J Nurs Midwifery. 2005;7(1):17–22. . Language: English. Entry Date: 20061027. Revision Date: 20150820. Publication Type: Journal Article.

[47] Gray WK, Dewhurst F, Dewhurst MJ, Orega G, Kissima J, Chaote P, et al Rates and predictors of three-year mortality in older people in rural Tanzania. Arch Gerontol Geriatr. 2016;62:36–42. 10.1016/j.archger.2015.10.008 . 26549489

[48] Guerchet M, Mouanga AM, M'Belesso P, Tabo A, Bandzouzi B, Paraïso MN, et al Factors Associated with Dementia Among Elderly People Living in Two Cities in Central Africa: The EDAC Multicenter Study. J Alzheimers Dis. 2012;29(1):15–24. 10.3233/JAD-2011-111364 . 22204904

[49] Hammami S, Mehri S, Hajem S, Koubaa N, Frih MA, Kammoun S, et al Awareness, treatment and control of hypertension among the elderly living in their home in Tunisia. BMC Cardiovasc Disord. 2011;11:65 10.1186/1471-2261-11-65 22044442 PMC3234182

[50] Hien H, Berthe A, Drabo MK, Meda N, Konate B, Tou F, et al Prevalence and patterns of multimorbidity among the elderly in Burkina Faso: cross-sectional study. Trop Med Int Health. 2014;19(11):1328–33. 10.1111/tmi.12377 25164626

[51] Ice GH, Zidron A, Juma E. Health and health perceptions among Kenyan grandparents. J Cross Cult Gerontol. 2008;23(2):111–29. 10.1007/s10823-008-9063-9 18437546

[52] Iribhogbe I. A Community Based Interventional Study on Health Status of Aged People in a Semi-Urban Community in South-South Nigeria. Int J Community Res. 2013;2(2):22–7.

[53] Ivy A, Tam J, Dewhurst MJ, Gray WK, Chaote P, Rogathi J, et al Ambulatory Blood Pressure Monitoring to Assess the White-Coat Effect in an Elderly East African Population. J Clin Hypertens (Greenwich). 2015;17(5):389–94. 10.1111/jch.12501 . 25690267 PMC8032030

[54] Kinyanda E, Kuteesa M, Scholten F, Mugisha J, Baisley K, Seeley J. Risk of major depressive disorder among older persons living in HIV-endemic central and southwestern Uganda. AIDS Care. 2016;28(12):1516–21. 10.1080/09540121.2016.1191601 2016-48426-005. 27263868

[55] Koopman JJE, van Bodegom D, Jukema JW, Westendorp RGJ. Risk of Cardiovascular Disease in a Traditional African Population with a High Infectious Load: A Population-Based Study. PLoS One. 2012;7(10):1–8. 10.1371/journal.pone.0046855 . PMC3469578 23071653

[56] Lasisi AO, Abiona T, Gureje O. Tinnitus in the elderly: Profile, correlates, and impact in the Nigerian study of ageing. Otolaryngol Head Neck Surg. 2010;143(4):510–5. 10.1016/j.otohns.2010.06.817 20869560

[57] Macia E, Duboz P, Gueye L. Prevalence, awareness, treatment and control of hypertension among adults 50 years and older in Dakar, Senegal. Cardiovasc J Afr. 2012;23(5):265–9. 10.5830/CVJA-2011-039 22002461 PMC3721830

[58] Mathenge W, Foster A, Kuper H. Urbanization, ethnicity and cardiovascular risk in a population in transition in Nakuru, Kenya: a population-based survey. BMC Public Health. 2010;10(1):569–80. 10.1186/1471-2458-10-569 . 20860807 PMC2956724

[59] Minicuci N, Biritwum RB, Mensah G, Yawson AE, Naidoo N, Chatterji S, et al Sociodemographic and socioeconomic patterns of chronic non-communicable disease among the older adult population in Ghana. Glob Health Action. 2014;7:21292 10.3402/gha.v7.21292 24746141 PMC3991840

[60] Mkhize X, Napier C, Oldewage-Theron W. The nutrition situation of free-living elderly in Umlazi township, South Africa. Health SA Gesondheid. 2013;18(1):1–8. 10.4102/hsag.v18i1.656

[61] Mugisha JO, Baisley K, Asiki G, Seeley J, Kuper H. Prevalence, Types, Risk Factors and Clinical Correlates of Anaemia in Older People in a Rural Ugandan Population. PLoS One. 2013;8(10):1–. 10.1371/journal.pone.0078394 . PMC3806814 24194926

[62] Nuertey BD, Alhassan AI, Nuertey AD, Mensah IA, Adongo V, Kabutey C, et al Prevalence of obesity and overweight and its associated factors among registered pensioners in Ghana; a cross sectional studies. BMC Obes. 2017;4:1–12. 10.1186/s40608-016-0139-8 PubMed PMID: 124088486. 28690855 PMC5496418

[63] Ochayi B, Thacher T. Risk factors for dementia in central Nigeria. Aging Ment Health. 2006;10(6):616–20. 10.1080/13607860600736182 17050090

[64] Ogunniyi A, Lane KA, Baiyewu O, Gao S, Gureje O, Unverzagt FW, et al Hypertension and incident dementia in community-dwelling elderly Yoruba Nigerians. Acta Neurol Scand. 2011;124(6):396–402. 10.1111/j.1600-0404.2011.01491.x 21303353 PMC3099146

[65] Osman A-Q. Nutrition & Health Status, Quality of Life, and Associated Factors among Non-Institutionalized Older Ghanaians [MPhil in Human Nutrition and Dietetics]. Kumasi: Kwame Nkrumah University of Science and Technology; 2017.

[66] Paddick SM, Kisoli A, Samuel M, Higginson J, Gray WK, Dotchin CL, et al Mild cognitive impairment in rural Tanzania: Prevalence, profile, and outcomes at 4-year follow-up. Am J Geriatr Psychiatry. 2015;23(9):950–9. 10.1016/j.jagp.2014.12.005 25579049

[67] Peltzer K, Phaswana-Mafuya N. Hypertension and associated factors in older adults in South Africa. Cardiovasc J Afr. 2013;24(3):66–71. 10.5830/CVJA-2013-002. PMC3721893 23736129

[68] Pilleron S, Aboyans V, Mbelesso P, Ndamba-Bandzouzi B, Desormais I, Lacroix P, et al Prevalence, awareness, treatment, and control of hypertension in older people in Central Africa: the EPIDEMCA study. J Am Soc Hypertens. 2017;11(7):449–60. 10.1016/j.jash.2017.04.013 28551091

[69] Putnam HWI, Jones R, Rogathi J, Gray WK, Swai B, Dewhurst M, et al Hypertension in a resource-limited setting: Is it associated with end organ damage in older adults in rural Tanzania? J Clin Hypertens (Greenwich). 2018;20(2):217–24. 10.1111/jch.13187 . 29446219 PMC8031133

[70] Raji YR, Abiona T, Gureje O. Awareness of hypertension and its impact on blood pressure control among elderly Nigerians: report from the Ibadan Study of Aging. Pan Afr Med J. 2017;27:190 10.11604/pamj.2017.27.190.11682 28904715 PMC5579467

[71] Scholten F, Mugisha J, Seeley J, Kinyanda E, Nakubukwa S, Kowal P, et al Health and functional status among older people with HIV/AIDS in Uganda. BMC Public Health. 2011;11:886 10.1186/1471-2458-11-886 22111659 PMC3256234

[72] Tianyi FL, Agbor VN, Njamnshi AKJHSR. Prevalence, awareness, treatment, and control of hypertension in Cameroonians aged 50 years and older: A community‐based study. Health Sci Rep. 2018;1(5):e44 10.1002/hsr2.44 30623073 PMC6266375

[73] Yerly P, Madeleine G, Riesen W, Bovet P. Low prevalence of abdominal aortic aneurysm in the Seychelles population aged 50 to 65 years. Cardiovasc J Afr. 2013;24(2):17–8. Epub 2013/04/25. 10.5830/CVJA-2012-070 23612948 PMC3734875

[74] Mkhize XN. Situation analysis of free-living elderly in Umlazi township. Durban: Durban University of Technology; 2011.

[75] Phaswana-Mafuya N, Peltzer K, Chirinda W, Musekiwa A, Kose Z. Sociodemographic predictors of multiple non-communicable disease risk factors among older adults in South Africa. Glob Health Action. 2013;6(1):20680 10.3402/gha.v6i0.20680. 24044582 PMC3776324

[76] Lloyd-Sherlock P, Beard J, Minicuci N, Ebrahim S, Chatterji S. Hypertension among older adults in low and middle-income countries: Prevalence, awareness and control. Int J Epidemiol. 2014;43(1):116–28. 10.1093/ije/dyt215 24505082 PMC3937973

[77] Lloyd-Sherlock P, Minicuci N, Corso B, Beard J, Chatterji S, Ebrahim S. Diseases of the Rich? The Social Patterning of Hypertension in Six Low- and Middle-Income Countries. Eur J Dev Res. 2017;29(4):827–42. 10.1057/s41287-016-0063-2 .

[78] Boateng GO, Adams EA, Odei Boateng M, Luginaah IN, Taabazuing M-M. Obesity and the burden of health risks among the elderly in Ghana: A population study. PLoS One. 2017;12(11):1–25. 10.1371/journal.pone.0186947 . PMC5695605 29117264

[79] Tyrovolas S, Koyanagi A, Garin N, Olaya B, Ayuso-Mateos JL, Miret M, et al Determinants of the components of arterial pressure among older adults–The role of anthropometric and clinical factors: A multi-continent study. Atherosclerosis. 2015;238(2):240–9. 10.1016/j.atherosclerosis.2014.11.029 25528433

[80] Adeloye D, Basquill C, Aderemi AV, Thompson JY, Obi FA. An estimate of the prevalence of hypertension in Nigeria: a systematic review and meta-analysis. J Hypertens. 2015;33(2):230–42. 10.1097/HJH.0000000000000413 25380154

[81] Schutte AE, Botha S, Fourie CMT, Gafane-Matemane LF, Kruger R, Lammertyn L, et al Recent advances in understanding hypertension development in sub-Saharan Africa. J Hum Hypertens. 2017;31(8):491 10.1038/jhh.2017.18 28332510

[82] Okpechi IG, Chukwuonye II, Tiffin N, Madukwe OO, Onyeonoro UU, Umeizudike TI, et al Blood pressure gradients and cardiovascular risk factors in urban and rural populations in Abia State South Eastern Nigeria using the WHO STEPwise approach. PLoS One. 2013;8(9):e73403 Epub 2013/09/17. 10.1371/journal.pone.0073403 24039932 PMC3764162

[83] Bosu WK. An overview of the nutrition transition in West Africa: implications for non-communicable diseases. Proc Nutr Soc. 2015;74(4):466–77. Epub 2014/12/23. 10.1017/S0029665114001669 . 25529539

[84] NCD Risk Factor Collaboration (NCD-RisC). Trends in adult body-mass index in 200 countries from 1975 to 2014: a pooled analysis of 1698 population-based measurement studies with 19.2 million participants. Lancet (London, England). 2016;387(10026):1377–96. Epub 2016/04/27. 10.1016/s0140-6736(16)30054-x . 27115820 PMC7615134

[85] Basu S, Millett C. Social epidemiology of hypertension in middle-income countries: determinants of prevalence, diagnosis, treatment, and control in the WHO SAGE study. Hypertension. 2013;62(1):18–26. Epub 2013/05/15. 10.1161/HYPERTENSIONAHA.113.01374 . 23670299

[86] Bosu WK. Determinants of Mean Blood Pressure and Hypertension among Workers in West Africa. Int J Hypertens. 2016;2016:3192149 Epub 2016/03/08. 10.1155/2016/3192149 26949543 PMC4754493

[87] Boateng GO, Adams EA, Odei Boateng M, Luginaah IN, Taabazuing MM. Obesity and the burden of health risks among the elderly in Ghana: A population study. PLoS One. 2017;12(11):e0186947 Epub 2017/11/09. 10.1371/journal.pone.0186947 29117264 PMC5695605

[88] Twagirumukiza M, De Bacquer D, Kips JG, de Backer G, Stichele RV, Van Bortel LM. Current and projected prevalence of arterial hypertension in sub-Saharan Africa by sex, age and habitat: an estimate from population studies. J Hypertens. 2011;29(7):1243–52. Epub 2011/05/05. 10.1097/HJH.0b013e328346995d . 21540748

[89] NCD Risk Factor Collaboration (NCD-RisC). Worldwide trends in blood pressure from 1975 to 2015: a pooled analysis of 1479 population-based measurement studies with 19.1 million participants. Lancet (London, England). 2017;389(10064):37–55. Epub 2016/11/20. 10.1016/s0140-6736(16)31919-5 27863813 PMC5220163

[90] Bovet P, Romain S, Shamlaye C, Mendis S, Darioli R, Riesen W, et al Divergent fifteen-year trends in traditional and cardiometabolic risk factors of cardiovascular diseases in the Seychelles. Cardiovasc Diabetol. 2009;8:34 Epub 2009/06/30. 10.1186/1475-2840-8-34 19558646 PMC2719584

[91] Laouani CK, Hmouda H, Ben MN, Ghannem H, Toumi S, Ajmi F. High blood presure for people aged more than 60 years in the distrct of Sousse. Tunis Med. 2004;82(11):1001–5. 15822468

[92] Macia E, Gueye L, Duboz P. Hypertension and Obesity in Dakar, Senegal. PLoS One. 2016;11(9):e0161544–e. 10.1371/journal.pone.0161544 . 27622534 PMC5021383

[93] Amugsi DA, Dimbuene ZT, Asiki G, Kyobutungi C. Quantile regression analysis of modifiable and non-modifiable drivers’ of blood pressure among urban and rural women in Ghana. Sci Rep. 2018;8(1):8515 10.1038/s41598-018-26991-4 29867184 PMC5986854

[94] Villareal DT, Miller BV III, Banks M, Fontana L, Sinacore DR, Klein S. Effect of lifestyle intervention on metabolic coronary heart disease risk factors in obese older adults.(Cardiovascular disease risk)(Author abstract). Am J Clin Nutr. 2006;84(6):1317 10.1093/ajcn/84.6.1317 17158411

[95] Amugsi DA, Dimbuene ZT, Mberu B, Muthuri S, Ezeh AC. Prevalence and time trends in overweight and obesity among urban women: an analysis of demographic and health surveys data from 24 African countries, 1991–2014. BMJ Open. 2017;7(10):e017344 Epub 2017/10/29. 10.1136/bmjopen-2017-017344 29079606 PMC5665233

[96] Ezejimofor M, Uthman O, Chen YF, Ezejimofor B, Ezeabasili A, Stranges S, et al Magnitude and pattern of hypertension in the Niger Delta: a systematic review and meta-analysis of community-based studies. J Glob Health. 2018;8(1):010420 Epub 2018/06/15. 10.7189/jogh.08.010420 29899980 PMC5997369

[97] Callender T, Woodward M, Roth G, Farzadfar F, Lemarie JC, Gicquel S, et al Heart failure care in low- and middle-income countries: a systematic review and meta-analysis. PLoS Med. 2014;11(8):e1001699 Epub 2014/08/15. 10.1371/journal.pmed.1001699 25117081 PMC4130667

[98] El-Tallawy HN, Farghaly WM, Rageh TA, Shehata GA, Abdel Hakeem M N, Badry R, et al Prevalence of trigeminal neuralgia in Al-Quseir city (Red sea Governorate), Egypt. Clin Neurol Neurosurg. 2013;115(9):1792–4. 10.1016/j.clineuro.2013.04.014 23692870

[99] World Health Organization. Active ageing: A policy framework. Geneva: WHO, 2002. 12040973

