# Supplementary material for: Hypertension in older adults in Africa: A systematic review and meta-analysis
Source: PLoS One. 2019 Apr 5;14(4):e0214934. doi: 10.1371/journal.pone.0214934 (PMC6450645; doi:10.1371/journal.pone.0214934)
Supplement: S3 Table — (DOCX) [file pone.0214934.s003.docx]

**S3 Table. List of excluded full text assessed for eligibility and the reasons for their exclusion**

| # | Author (Year) | Title | Reason |
| --- | --- | --- | --- |
| 1 | Abd Elaziz 2015 | Prevalence of metabolic syndrome and cardiovascular risk factors among voluntary screened middle-aged and elderly Egyptians | Hospital-based study |
| 2 | Adebiyi 2016 | Cognitive impairment among the aging population in a community in southwest Nigeria. | No prevalence of hypertension estimate |
| 3 | Bachir Cherif 2016 | The characteristics of arterial hypertension in postmenopausal women in the area of Blida (Algeria) | Wrong age group |
| 4 | Benghanem Gharbi 2016 | Chronic kidney disease, hypertension, diabetes, and obesity in the adult population of Morocco: how to avoid "over"- and "under"-diagnosis of CKD | Wrong age group |
| 5 | Boateng 2015 | Examining the risk factors associated with hypertension among the elderly in Ghana. | Self-reported hypertension |
| 6 | Chan 2017 | Is trusting others related to better health? An investigation of older adults across six non-Western countries. | Self-reported hypertension |
| 7 | Clausen 2000 | Morbidity and health care utilisation among elderly people in Mmankgodi village, Botswana | Different threshold for hypertension |
| 8 | Clausen 2005 | Chronic diseases and health inequalities in older persons in Botswana (southern Africa): a national survey | Different threshold for hypertension |
| 9 | Coumé 2012 | Estimation de la prévalence du déficit cognitif dans une population de personnes âgées sénégalaises du Centre médico-social et universitaire de l’Institution de prévoyance retraite du Sénégal. = Estimate of the prevalence of cognitive impairment in an elderly population of the health center of Senegalese national retirement institution | Hospital-based study |
| 10 | Doszhanova 2015 | Health of elderly people in the context of their profession | Self-reported hypertension |
| 11 | El-Sherbiny 2016 | A comprehensive assessment of the physical, nutritional, and psychological health status of the elderly populace in the Fayoum Governorate (Egypt) | Self-reported hypertension |
| 12 | Ezenwaka 1997 | The prevalence of insulin resistance and other cardiovascular disease risk factors in healthy elderly southwestern Nigerians | Different threshold for hypertension |
| 13 | Gradidge 2017 | Factors associated with obesity and metabolic syndrome in ageing black South African women | No prevalence of hypertension estimated |
| 14 | Grobler 2012 | Cardiovascular risk of an elderly, black South African population in Sharpeville, South Africa | Institutionalized subjects |
| 15 | Gureje 2009 | Profile, comorbidity and impact of insomnia in the Ibadan study of ageing | Self-reported hypertension |
| 16 | Gureje 2008 | Depression and disability: comparisons with common physical conditions in the Ibadan study of aging | Self-reported hypertension |
| 17 | Ice 2010 | The impact of caregiving on the health and well-being of Kenyan Luo grandparents. | No prevalence of hypertension estimated |
| 18 | Kadiri 1997 | Cardiovascular risk factors in middle aged Nigerians | Different threshold for hypertension |
| 19 | Kailembo 2016 | Common risk factors and edentulism in adults, aged 50 years and over, in China, Ghana, India and South Africa: results from the WHO Study on global AGEing and adult health (SAGE) | Self-reported hypertension |
| 20 | Kowal 2012 | Hypertension in developing countries | Outside the African region |
| 21 | Kunna 2017 | Measurement and decomposition of socioeconomic inequality in single and multimorbidity in older adults in China and Ghana: Results from the WHO study on global AGEing and adult health (SAGE) | No prevalence of hypertension estimated |
| 22 | Kuven 2011 | The prevalence, management and follow-up of diabetes mellitus and its relationship to hypertension and obesity in persons aged 60 years and over living in a peri-urban area of South Africa | Self-reported hypertension |
| 23 | Laouani Kechrid 2004 | High blood presure for people aged more than 60 years in the distrct of Sousse | Hospital-based study |
| 24 | Lasisi 2010 | The prevalence and correlates of self-reported hearing impairment in the Ibadan Study of Ageing | Self-reported hypertension |
| 25 | Lasisi 2014 | Prevalence and correlates of dizziness in the Ibadan Study of Ageing | Self-reported hypertension |
| 26 | Laverty 2015 | Associations between active travel and weight, blood pressure and diabetes in six middle income countries: A cross-sectional study in older adults. | No prevalence of hypertension estimated |
| 27 | Menyanu 2017 | Salt Use Behaviours of Ghanaians and South Africans: A Comparative Study of Knowledge, Attitudes and Practices | Wrong age group |
| 28 | Mokgele 1999 | Health needs assessment of the elderly people of Mangaung. | Different threshold for hypertension |
| 29 | Nyirenda 2013 | Health, wellbeing, and disability among older people infected or affected by HIV in Uganda and South Africa. | Self-reported hypertension |
| 30 | Mwangengwa 2014 | Profile of plasma lipids and degree of derangements among the elderly of Morogoro region, Tanzania | No prevalence of hypertension estimated |
| 31 | Nwamarah 2014 | Fruit and vegetable consumption pattern and health challenges of elderly (>=60 years) staff in the university of Nigeria, Nsukka and Enugu Campuses: A case study | Self-reported hypertension |
| 32 | Oldewage-Theron 2008 | Health status of an elderly population in Sharpeville, South Africa. | Different threshold for hypertension |
| 33 | Otitoola 2013 | A cohort study on the prevalence of obesity and hypertension among sharpeville elderly in South Africa (2005-2011) | No prevalence of hypertension estimated |
| 34 | Temmar 2013 | Elderly Algerian women lose their sex-advantage in terms of arterial stiffness and cardiovascular profile | No prevalence of hypertension estimated |
| 35 | van Vuuren 2009 | Effectiveness of influenza vaccination in the elderly in South Africa | Hospital-based study |
| 36 | Vellakkal 2015 | Are estimates of socioeconomic inequalities in chronic disease artefactually narrowed by self-reported measures of prevalence in low-income and middle-income countries? Findings from the WHO-SAGE survey | Wrong age group |
| 37 | Ware 2017 | Associations between dietary salt, potassium and blood pressure in South African adults: WHO SAGE Wave 2 Salt & Tobacco | Wrong age group |
| 38 | Werfalli 2016 | Health related quality of life and functional disability among South African older adults with diabetes: results from study on global ageing and adult health (SAGE) | No prevalence of hypertension estimated |
| 39 | Yawson 2014 | Self-reported cataracts in older adults in Ghana: sociodemographic and health related factors | Self-reported hypertension |
| 40 | Yawson 2013 | Tobacco use in older adults in Ghana: sociodemographic characteristics, health risks and subjective wellbeing | Self-reported hypertension |
