# Supplementary material for: Hypertension in older adults in Africa: A systematic review and meta-analysis
Source: PLoS One. 2019 Apr 5;14(4):e0214934. doi: 10.1371/journal.pone.0214934 (PMC6450645; doi:10.1371/journal.pone.0214934)
Supplement: S4 Table — (DOCX) [file pone.0214934.s004.docx]

**S4 Table. List of multiple papers on the same study subjects and their primary studies**

| # | Author (Year) | Title | Primary study | Ref # of primary study in bibliography |
| --- | --- | --- | --- | --- |
| 1 | Dewhurst 2012 | Strikingly low prevalence of atrial fibrillation in elderly Tanzanians | Dewhurst 2013 | [44] |
| 2 | Dewhurst 2011 | Hypertension in Tanzanian elderly: the disparity between prevalence, detection and treatment-a rule of sixths? | Dewhurst 2013 | [44] |
| 3 | Dewhurst 2013 | The Prevalence and Clinical Correlates of Atrial Fibrillation in Those Aged 70 and Over in the Hai District of Northern Tanzania | Dewhurst 2013 | [44] |
| 4 | El Tallawy 2014 | Epidemiological and clinical profile of Alzheimer's dementia in Upper Egypt | El Tallawy 2012 | [46] |
| 5 | Farghaly 2014 | Prevalence of vascular dementia in Upper Egypt | El Tallawy 2012 | [46] |
| 6 | Guerchet 2012 | Epidemiology of peripheral artery disease in elder general population of two cities of central Africa: Bangui and Brazzaville | Guerchet 2012 | [39] |
| 7 | Mbelesso 2012 | Épidémiologie des démences chez les personnes âgées dans le troisième arrondissement de la ville de Bangui (République Centrafricaine) | Guerchet 2012 | [39] |
| 8 | Hammami 2015 | Hypertension and disability in Tunisian's elderly | Hammami 2011 | [49] |
| 9 | Hammami 2012 | Prevalence of diabetes mellitus among non institutionalized elderly in Monastir City | Hammami 2011 | [49] |
| 10 | Koopman 2014 | Scarcity of atrial fibrillation in a traditional African population: A community-based study | Koopman 2012 | [55] |
| 11 | Boateng 2017 | Obesity and the burden of health risks among the elderly in Ghana: A population study. | Minicuci 2014 | [58] |
| 12 | Mkhize 2011 | Situation analysis of free-living elderly in Umlazi township | Mkhize 2013 | [59] |
| 13 | Lloyd-Sherlock 2014 | Pensions and the Health of Older People in South Africa: Is there an Effect? | Peltzer 2013 | [41] |
| 14 | Peltzer 2012 | Sociodemographic and health correlates of sleep problems and duration in older adults in South Africa | Peltzer 2013 | [41] |
| 15 | Peltzer 2013 | Depression and associated factors in older adults in South Africa | Peltzer 2013 | [41] |
| 16 | Peltzer 2012 | Cognitive functioning and associated factors in older adults in South Africa. | Peltzer 2013 | [41] |
| 17 | Peltzer 2013 | Arthritis and associated factors in older adults in South Africa | Peltzer 2013 | [41] |
| 18 | Peltzer 2012 | Fruit and vegetable intake and associated factors in older adults in South Africa | Peltzer 2013 | [41] |
| 19 | Waterhouse 2017 | The impact of multi-morbidity on disability among older adults in South Africa: do hypertension and socio-demographic characteristics matter? | Peltzer 2013 | [41] |
| 20 | Phaswana-Mafuya 2013 | Sociodemographic predictors of multiple non-communicable disease risk factors among older adults in South Africa | Peltzer 2013 | [41] |
| 21 | Lloyd-Sherlock 2017 | Diseases of the Rich? The Social Patterning of Hypertension in Six Low- and Middle-Income Countries. | Peltzer 2013; Minicuci 2014 | [41, 58] |
| 22 | Arokiasamy 2017 | Chronic noncommunicable diseases in 6 low- and middle-income countries: Findings from wave 1 of the world health organization's Study on Global Ageing and Adult Health (SAGE) | Peltzer 2013; Minicuci 2014 | [41, 58] |
| 23 | Koyanagi 2014 | Chronic conditions and sleep problems among adults aged 50 years or over in nine countries: A multi-country study | Peltzer 2013; Minicuci 2014 | [41, 58] |
| 24 | Maurer 2015 | One-year routine opportunistic screening for hypertension in formal medical settings and potential improvements in hypertension awareness among older persons in developing countries: Evidence from the study on global ageing and adult health (SAGE) | Peltzer 2013; Minicuci 2014 | [41, 58] |
| 25 | Tyrovolas 2015 | Determinants of the components of arterial pressure among older adults - The role of anthropometric and clinical factors: A multi-continent study | Peltzer 2013; Minicuci 2014 | [41, 58] |
| 26 | Wu 2015 | Common risk factors for chronic non-communicable diseases among older adults in China, Ghana, Mexico, India, Russia and South Africa: the study on global AGEing and adult health (SAGE) wave 1 | Peltzer 2013; Minicuci 2014 | [41, 58] |
| 27 | Yang 2016 | Prevalence, awareness, treatment, and control of hypertension in the older population: results from the multiple national studies on ageing | Peltzer 2013; Minicuci 2014 | [41, 58] |
| 28 | Pilleron 2016 | Particularities of the epidemiology of hypertension in the elderly in Central Africa: The EPIDEMCA study | Pilleron 2017 | [38] |
| 29 | Putnam 2017 | Hypertensive end organ damage in an elderly cohort of tanzanians | Putnam 2018 | [36] |
