# Supplementary material for: Hypertension in older adults in Africa: A systematic review and meta-analysis
Source: PLoS One. 2019 Apr 5;14(4):e0214934. doi: 10.1371/journal.pone.0214934 (PMC6450645; doi:10.1371/journal.pone.0214934)
Supplement: S5 Table — (DOCX) [file pone.0214934.s005.docx]

**S5 Table. Evaluation of bias of included studies based on the Hoy criteria**

| # | Primary Reference | *Q1* | *Q2* | *Q3* | *Q4* | *Q5* | *Q6* | *Q7* | *Q8* | *Q9* | *Q10* | *Overall risk of study bias* |
| --- | --- | --- | --- | --- | --- | --- | --- | --- | --- | --- | --- | --- |
| 1 | Tianyi 2017 | Low risk | Low risk | Low risk | Low risk | Low risk | Low risk | Low risk | Low risk | Low risk | Low risk | **Low risk** |
| 2 | Pilleron 2017A | Low risk | Low risk | Low risk | Low risk | Low risk | Low risk | Low risk | Low risk | Low risk | Low risk | **Low risk** |
| 3 | Pilleron 2017B | Low risk | Low risk | Low risk | Low risk | Low risk | Low risk | Low risk | Low risk | Low risk | Low risk | **Low risk** |
| 4 | Guerchet 2012A | Low risk | Low risk | Low risk | Low risk | Low risk | Low risk | Low risk | Low risk | Low risk | Low risk | **Low risk** |
| 5 | Guerchet 2012B | Low risk | Low risk | Low risk | Low risk | Low risk | Low risk | Low risk | Low risk | Low risk | Low risk | **Low risk** |
| 6 | Mathenge 2010 | Low risk | Low risk | Low risk | Low risk | Low risk | Low risk | Low risk | Low risk | Low risk | Low risk | **Low risk** |
| 7 | Ice 2008 | High risk | High risk |  | Low risk | Low risk | Low risk | Low risk | Low risk | Low risk | Low risk | **High risk** |
| 8 | Yerly 2013 | Low risk | Low risk | Low risk | Low risk | Low risk | Low risk | Low risk | Low risk | Low risk | Low risk | **Low risk** |
| 9 | Dewhurst 2013 | Low risk | Low risk | Low risk | Low risk | Low risk | Low risk | Low risk | Low risk | Low risk | Low risk | **Low risk** |
| 10 | Ivy 2015 | Low risk | Low risk | Low risk | High risk | Low risk | Low risk | Low risk | Low risk | Low risk | Low risk | **Moderate risk** |
| 11 | Paddick 2015 | Low risk | High risk | Low risk | High risk | Low risk | Low risk | Low risk | Low risk | Low risk | Low risk | **High risk** |
| 12 | Gray 2016 | Low risk | Low risk | Low risk | High risk | Low risk | Low risk | Low risk | Low risk | Low risk | Low risk | **Moderate risk** |
| 13 | Putnam 2018 | Low risk | Low risk | Low risk | High risk | Low risk | Low risk | Low risk | Low risk | Low risk | Low risk | **Moderate risk** |
| 14 | Mugisha 2013 | Low risk | Low risk | Low risk | Low risk | Low risk | Low risk | Low risk | Low risk | Low risk | Low risk | **Moderate risk** |
| 15 | Kinyanda 2016 | High risk | High risk | Low risk | Low risk | Low risk | Low risk | Low risk | Low risk | Low risk | Low risk | **High risk** |
| 16 | Scholten 2011 | Low risk | Low risk | Low risk | Low risk | Low risk | Low risk | Low risk | Low risk | Low risk | Low risk | **Low risk** |
| 17 | Chami 2015 | Low risk | Low risk | Low risk | Low risk | Low risk | Low risk | Low risk | Low risk | Low risk | Low risk | **Low risk** |
| 18 | El Tallawy 2012 | Low risk | Low risk | Low risk | Low risk | Low risk | Low risk | Low risk | Low risk | Low risk | Low risk | **Low risk** |
| 19 | Hammami 2011 | High risk | Low risk | Low risk | Low risk | Low risk | Low risk | Low risk | Low risk | Low risk | Low risk | **Low risk** |
| 20 | Mkhize 2013 | Low risk | Low risk | Low risk | Low risk | Low risk | Low risk | Low risk | Low risk | Low risk | Low risk | **Low risk** |
| 21 | Peltzer 2013 | Low risk | Low risk | Low risk | High risk | Low risk | Low risk | Low risk | Low risk | Low risk | Low risk | **Moderate risk** |
| 22 | Hien 2014 | Low risk | Low risk | Low risk | Low risk | Low risk | Low risk | Low risk | Low risk | Low risk | Low risk | **Low risk** |
| 23 | Minicuci 2014 | Low risk | Low risk | Low risk | Low risk | Low risk | Low risk | Low risk | Low risk | Low risk | Low risk | **Low risk** |
| 24 | Koopman 2012 | Low risk | Low risk | Low risk | Low risk | Low risk | Low risk | Low risk | Low risk | Low risk | Low risk | **Low risk** |
| 25 | Nuertey 2017 | High risk | High risk | Low risk | Low risk | Low risk | Low risk | Low risk | Low risk | Low risk | Low risk | **High risk** |
| 26 | Osman 2017 | High risk | High risk | Low risk | Low risk | Low risk | Low risk | Low risk | Low risk | Low risk | Low risk | **High risk** |
| 27 | Fajemilehin 2005 | High risk | High risk | High risk | Low risk | Low risk | Low risk | Low risk | Low risk | Low risk | Low risk | **High risk** |
| 28 | Ochayi 2006 | Low risk | Low risk | Low risk | Low risk | Low risk | Low risk | Low risk | Low risk | Low risk | Low risk | **Low risk** |
| 29 | Lasisi 2010 | Low risk | High risk | Low risk | Low risk | Low risk | Low risk | Low risk | Low risk | Low risk | Low risk | **Moderate risk** |
| 30 | Ogunniyi 2011A | Low risk | Low risk | Low risk | Low risk | Low risk | Low risk | Low risk | Low risk | Low risk | Low risk | **Low risk** |
| 31 | Ogunniyi 2011B | Low risk | Low risk | Low risk | High risk | Low risk | Low risk | Low risk | Low risk | Low risk | Low risk | **Moderate risk** |
| 32 | Raji 2017 | High risk | Low risk | Low risk | High risk | Low risk | Low risk | Low risk | Low risk | Low risk | Low risk | **Moderate risk** |
| 33 | Iribhogbe 2013 | High risk | High risk | High risk | Low risk | Low risk | Low risk | Low risk | Low risk | Low risk | Low risk | **High risk** |
| 34 | Abegunde 2013 | Low risk | Low risk | Low risk | Low risk | High risk | Low risk | Low risk | Low risk | Low risk | Low risk | **Low risk** |
| 35 | Macia 2012 | Low risk | Low risk | Low risk | High risk | Low risk | Low risk | Low risk | Low risk | Low risk | Low risk | **Moderate risk** |
| 36 | Duboz 2015 | High risk | High risk | High risk | High risk | Low risk | Low risk | Low risk | Low risk | Low risk | Low risk | **High risk** |
| 37 | Kamoun 2006 | Low risk | Low risk | Low risk | High risk | Low risk | Low risk | Low risk | Low risk | Low risk | Low risk | **Low risk** |

List of 10 questions (Q1 – Q10) applied to the studies:

1. *Was the study's target population a close representation of the national population in relation to relevant variables, e.g. age, sex, occupation?*
2. *Was the sampling frame a true or close representation of the target population?*
3. *Was some form of random selection used to select the sample, OR, was a census undertaken?*
4. *Was the likelihood of non-response bias minimal?*
5. *Were data collected directly from the subjects (as opposed to a proxy)?*
6. *Was an acceptable case definition used in the study?*
7. *Was the study instrument that measured the parameter of interest (e.g. prevalence of low back pain) shown to have reliability and validity (if necessary)?*
8. *Was the same mode of data collection used for all subjects?*
9. *Was the length of the shortest prevalence period for the parameter of interest appropriate?*
10. *Were the numerator(s) and denominator(s) for the parameter of interest appropriate?*
