# Supplementary material for: Hypertension in older adults in Africa: A systematic review and meta-analysis
Source: PLoS One. 2019 Apr 5;14(4):e0214934. doi: 10.1371/journal.pone.0214934 (PMC6450645; doi:10.1371/journal.pone.0214934)
Supplement: S6 Table — (DOCX) [file pone.0214934.s006.docx]

**S6 Table. Univariate and multivariate meta-regression exploring potential sources of heterogeneity in the prevalence of hypertension**

| Model | N | | Coefficient (95% CI) | Tau^2^ | Adj. R^2^ (%) | I^2^ (%) | p value |
| --- | --- | --- | --- | --- | --- | --- | --- |
| No covariates | | 37 | 0.561 (0.503, 0.618) | 0.026 |  | 95.2 | <0.001 |
| Univariate | |  |  |  |  |  |  |
| Study year | | 35 | .0049 (-0.0110, 0.0207) | 0.027 | -2.96 | 95.3 | 0.537 |
| Publication year | | 37 | 0.0121 (-0.0054, 0.0296) | 0.026 | 1.73 | 95.4 | 0.168 |
| Enrolled sample size | | 37 | 2.69e-6 (-4.22e-5, 4.75e-5) | 0.027 | -3.29 | 95.4 | 0.904 |
| Percentage obesity | | 17 | 0.0041 (0.0003, 0.0079) | 0.012 | 23.19 | 92.0 | 0.036 |
| Percentage no education | | 23 | 0.0006 (-0.0024, 0.0036) | 0.024 | -4.54 | 95.5 | 0.676 |
| Device (ref: manual type) | | 22 | 0.1317 (-0.0267, 0.2900) | 0.025 | 8.45 | 96.6 | 0.098 |
|  | |  |  |  |  |  |  |
| Multiple covariates | | 16 |  | 0.008 | 50.00 | 87.5 | 0.030 |
| Study year | | 16 | -0.0248 (-0.0470, -0.0027) |  |  |  | 0.031 |
| Publication year | | 16 | 0.0320 (0.0076, 0.0564) |  |  |  | 0.027 |
| Enrolled sample size | | 16 | -4.73e-6 (-4.09e-5, 3.14e-5) |  |  |  | 0.779 |
| Percentage obesity | | 16 | 0.0044 (0.0009, 0.0079) |  |  |  | 0.018 |
