# Supplementary figures and images for: Hypertension in older adults in Africa: A systematic review and meta-analysis
Source: PLoS One. 2019 Apr 5;14(4):e0214934. doi: 10.1371/journal.pone.0214934 (PMC6450645; doi:10.1371/journal.pone.0214934)

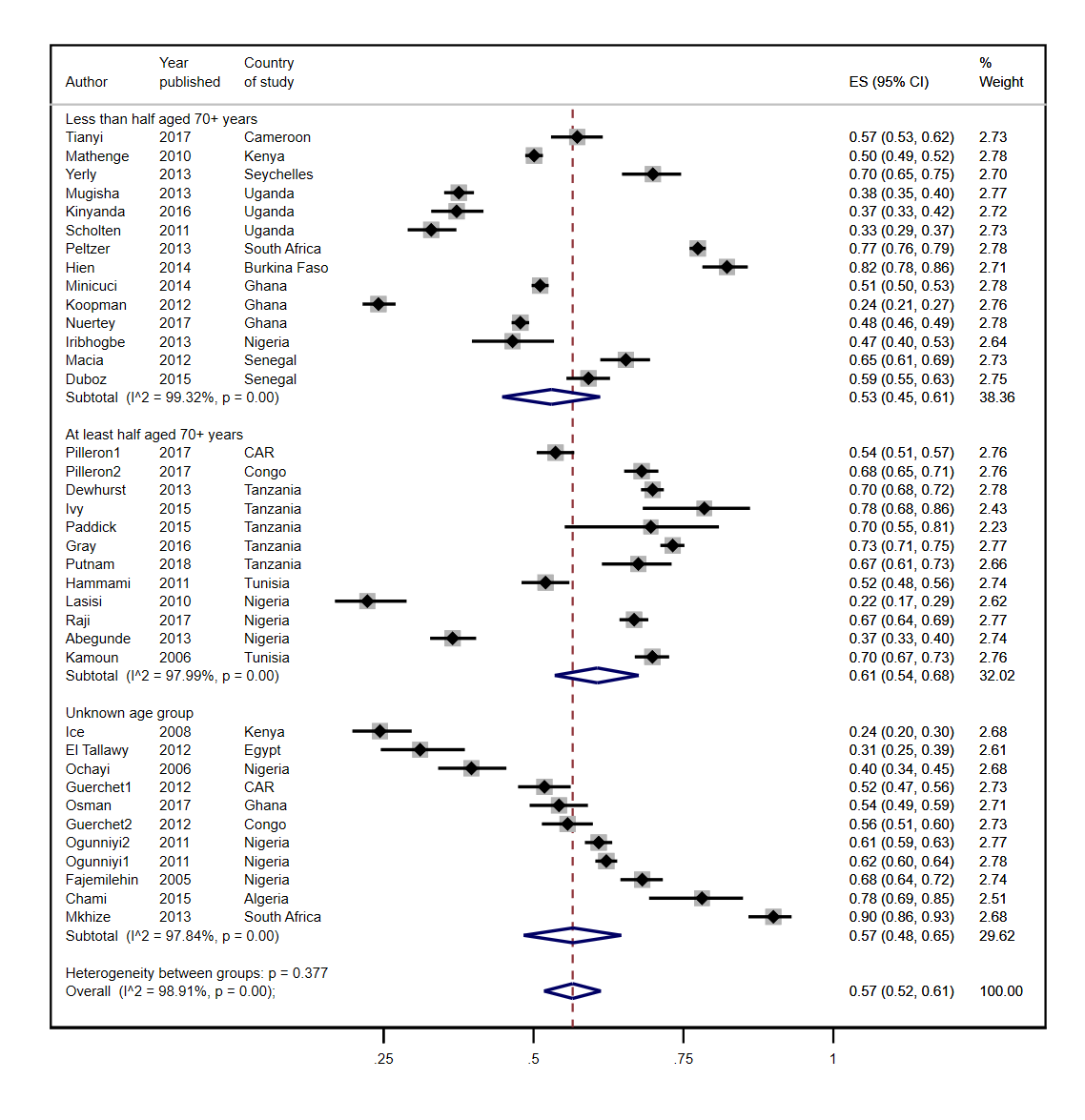

Supplement: S1 Fig — (TIF) [file pone.0214934.s007.tif]

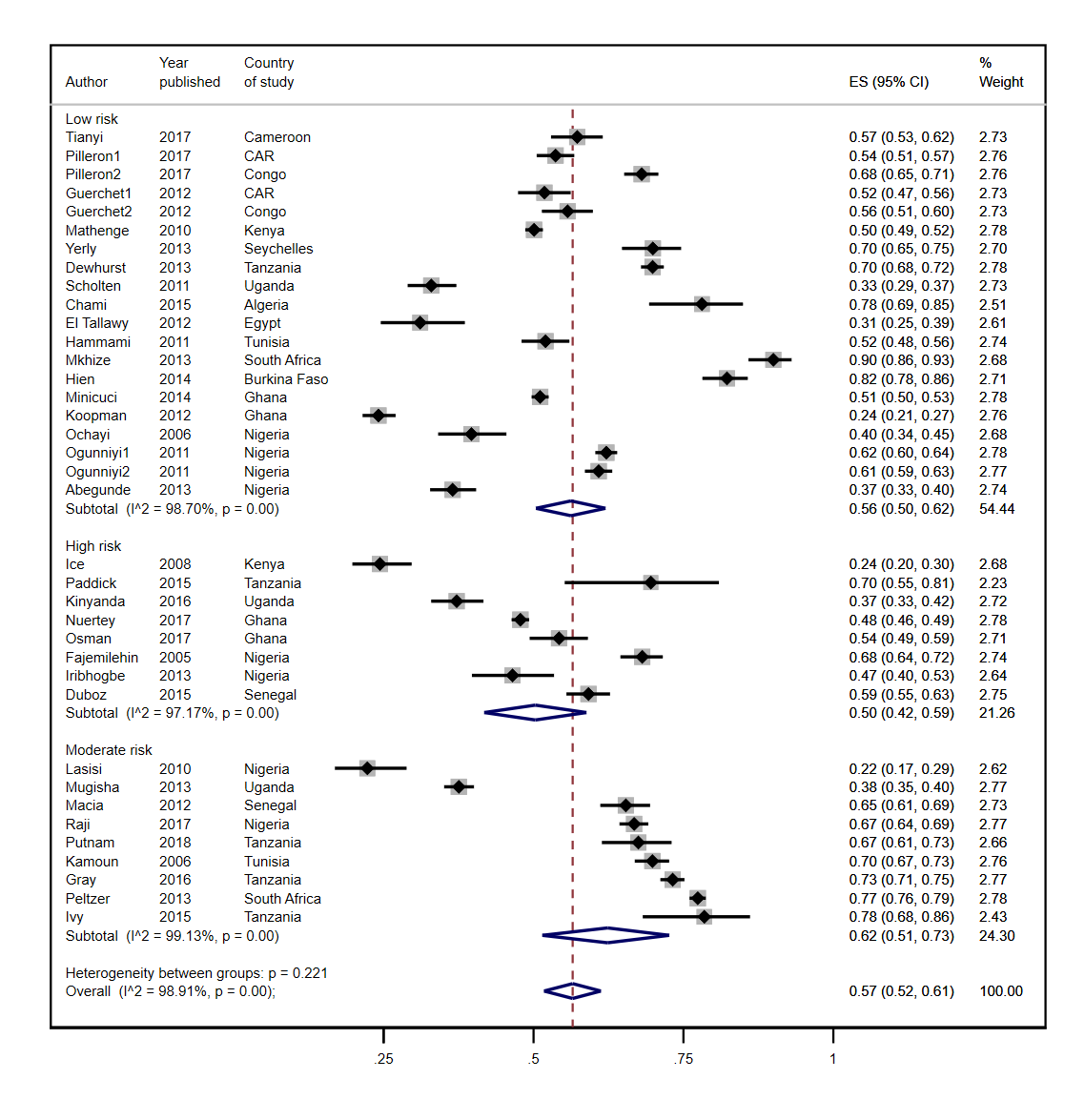

Supplement: S2 Fig — (TIF) [file pone.0214934.s008.tif]

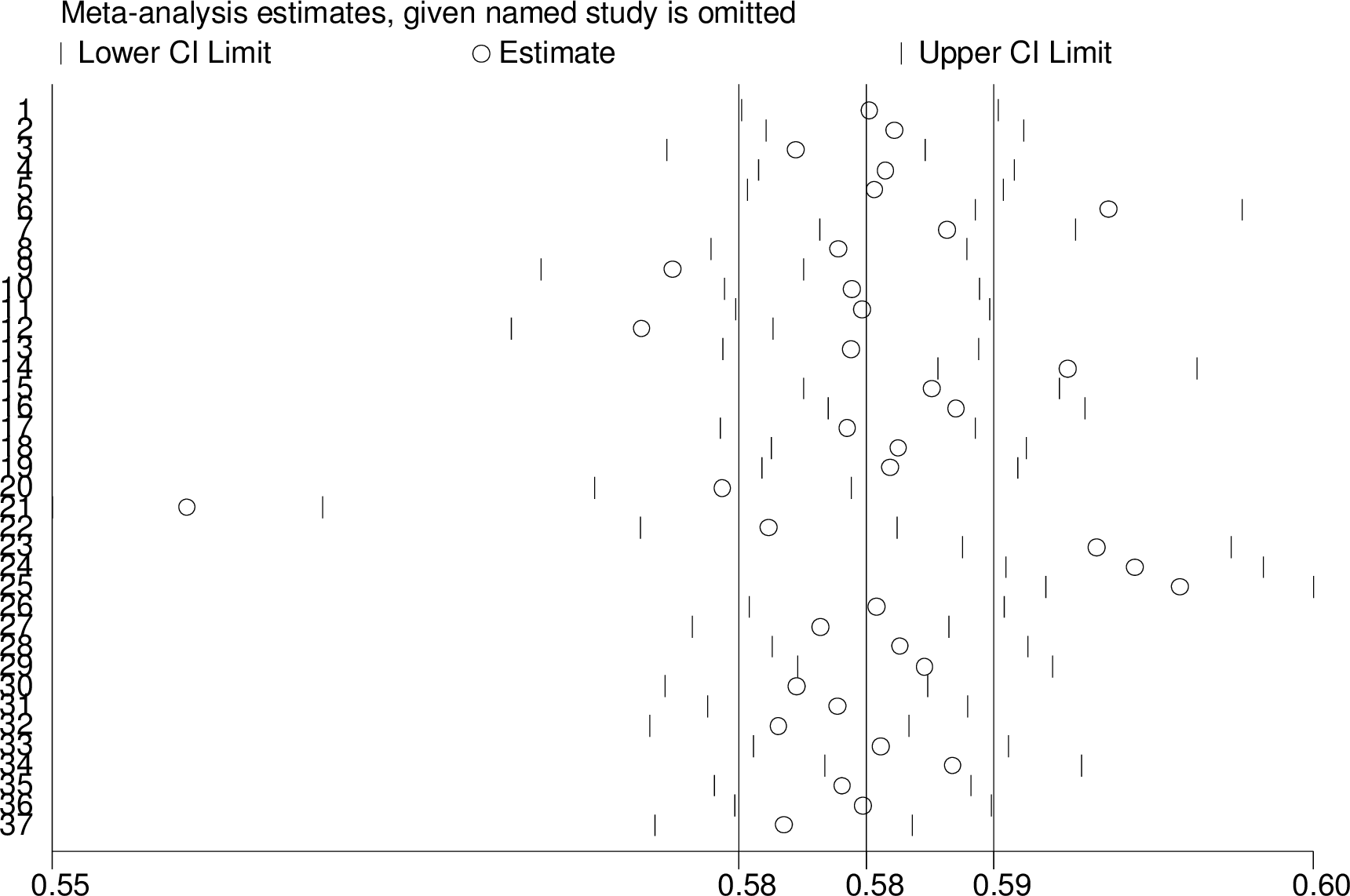

Supplement: S3 Fig — (TIF) [file pone.0214934.s009.tif]

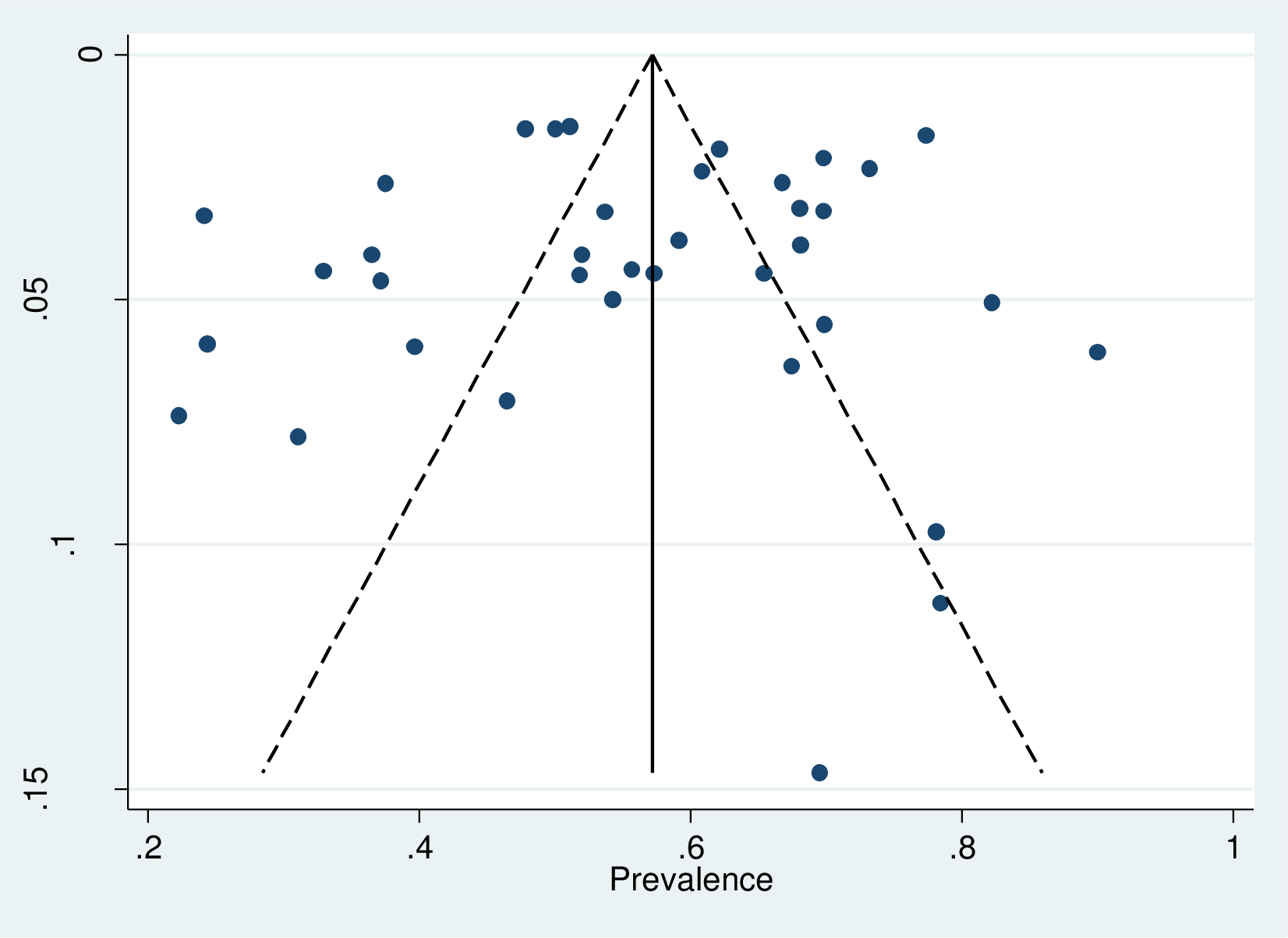

Supplement: S4 Fig — (TIF) [file pone.0214934.s010.tif]
